# Mononuclear manganese complexes as hydrogen evolving catalysts

**DOI:** 10.3389/fchem.2022.993085

**Published:** 2022-10-07

**Authors:** Vishakha Kaim, Meenakshi Joshi, Matthias Stein, Sandeep Kaur-Ghumaan

**Affiliations:** ^1^ Department of Chemistry, University of Delhi, Delhi, India; ^2^ Max-Planck-Institute for Dynamics of Complex Technical Systems, Molecular Simulations and Design Group, Magdeburg, Germany

**Keywords:** bioinorganic chemistry, manganese catalyst, hydrogen evolution, hydrogenase, redox activity, reaction mechanism, computational catalysis

## Abstract

Molecular hydrogen (H_2_) is one of the pillars of future non-fossil energy supply. In the quest for alternative, non-precious metal catalysts for hydrogen generation to replace platinum, biological systems such as the enzyme hydrogenase serve as a blueprint. By taking inspiration from the bio-system, mostly nickel- or iron-based catalysts were explored so far. Manganese is a known oxygen-reducing catalyst but has received much less attention for its ability to reduce protons in acidic media. Here, the synthesis, characterization, and reaction mechanisms of a series of four mono-nuclear Mn(I) complexes in terms of their catalytic performance are reported. The effect of the variation of equatorial and axial ligands in their first and second coordination spheres was assessed pertaining to their control of the turnover frequencies and overpotentials. All four complexes show reactivity and reduce protons in acidic media to release molecular hydrogen H_2_. Quantum chemical studies were able to assign and interpret spectral characterizations from UV–Vis and electrochemistry and rationalize the reaction mechanism. Two feasible reaction mechanisms of electrochemical (E) and protonation (C) steps were compared. Quantum chemical studies can assign peaks in the cyclic voltammetry to structural changes of the complex during the reaction. The first one-electron reduction is essential to generate an open ligand-based site for protonation. The distorted octahedral Mn complexes possess an inverted second one-electron redox potential which is a pre-requisite for a swift and facile release of molecular hydrogen. This series on manganese catalysts extends the range of elements of the periodic table which are able to catalyze the hydrogen evolution reaction and will be explored further.

## Introduction

Molecular hydrogen (H_2_) is one of the most-promising energy carriers in the future due to its zero-carbon combustion (Camack et al., 2001; Penner, 2005). The design and development of novel molecular catalytic systems for the hydrogen evolution reaction (HER) have received significant attention during the last decade. The electrocatalytic reduction of a proton to molecular hydrogen (H_2_) might help in the industrial process development, leaving behind fossil fuels, and toward a hydrogen economy. (Schlögl, 2013) Most current industrial processes rely on platinum as a highly efficient catalyst for hydrogen generation. However, there is an urgent need to substitute precious platinum with more abundant first-row transition metal catalysts that take inspiration from biological systems (Artero and Fontecave, 2005; Cracknell et al., 2008).

In global industrial processes, hydrogen is used on a massive scale, for example, in ammonia production (Haber–Bosch) or as a highly selective reducing agent in the production of active pharmaceutical ingredients (APIs), to name only a few. So far, hydrogen production is based on fossil fuels and/or highly efficient (precious) metal catalysts (Lubitz and Tumas, 2007). The processes are relatively energy-inefficient and lead to emission of significant quantities of greenhouse gases. Evolution has led to a family of enzymes, namely, the hydrogenases that use hydrogen as an energy carrier for their metabolism. Those hydrogenases have developed their own bio-inorganic transition metal catalysts to produce or utilize hydrogen as an energy source. They do so at ambient temperature and normal pressure and make use of the non-precious metals iron and nickel. Their active sites have inspired the synthesis of many bio-mimetic and bio-inspired catalysts (Evans and Pickett, 2003).

A direct comparison of a platinum catalyst with a hydrogenase equivalent has shown that the bio-hydrogen production by the enzyme occurs at rates comparable to those of electrodeposited platinum and with less susceptibility to CO poisoning (Jones et al., 2002). Since the density of the catalytic sites for the enzyme is a lot lower than that for platinum, the enzyme’s active site must turn over faster than a corresponding platinum atom to attain the same overall current. The performance of an electrode-immobilized nickel catalyst was directly compared to the performance of the [NiFe]-hydrogenase enzyme. At acidic pH and in the presence of carbon monoxide, the enzyme outperformed the synthetic catalyst and pointed toward the utilization of bio-inspired complexes in fuel cells (Rodriguez-Maciá et al., 2015).

**SCHEME 1:**
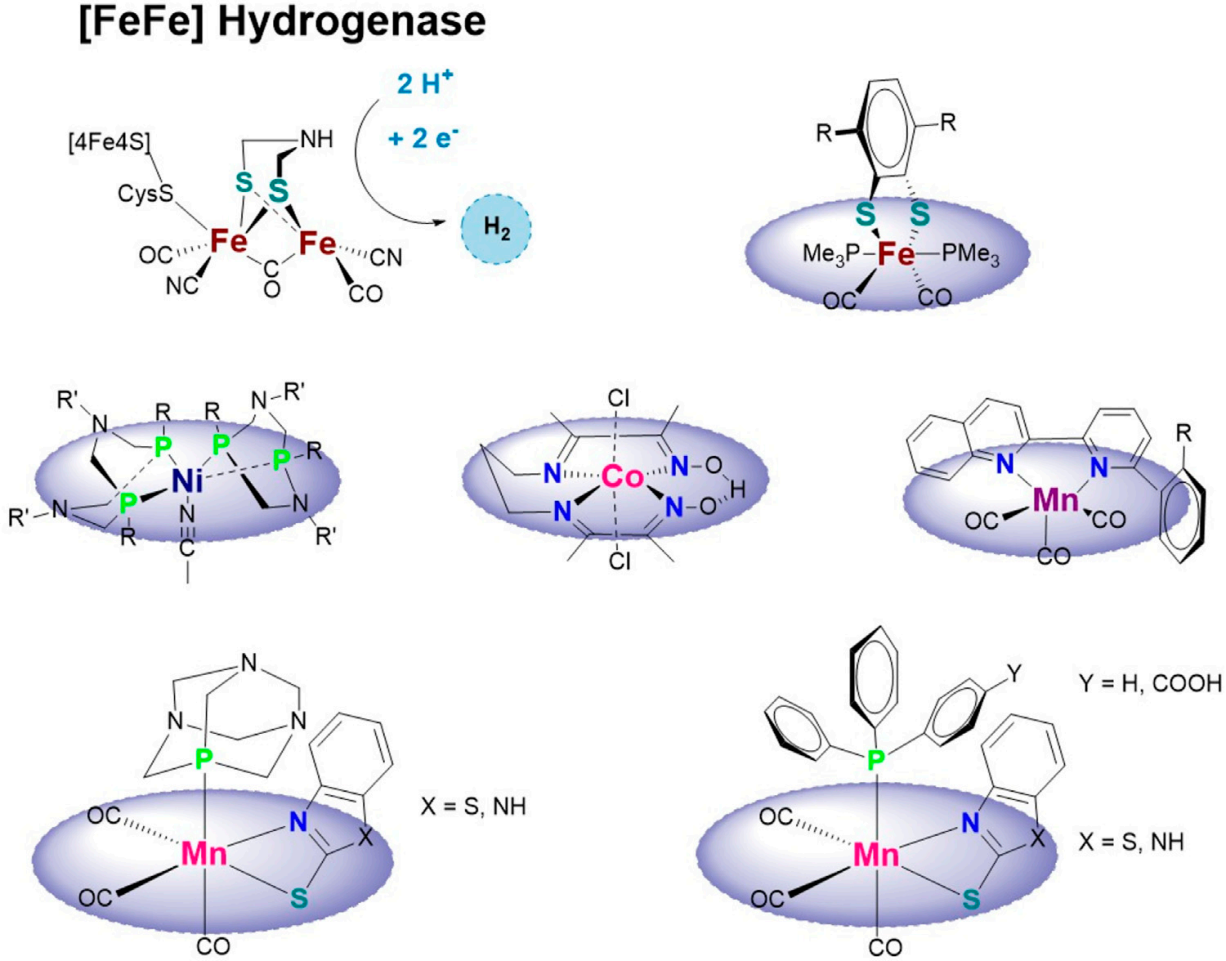
Active site of the [FeFe] hydrogenase enzyme and bio-inspired mono-nuclear hydrogen-evolving complexes based on iron (Kaur-Ghumaan et al., 2010), nickel (Wilson et al., 2006; Brazzolotto et al., 2016), cobalt (Fang et al., 2014), or manganese (Kaim and Kaur-Ghumaan, 2021; Yang et al., 2021) and this work.

Drawing inspiration from the naturally occurring [FeFe]-, [NiFe]-, and hydrogenase enzymes, a plethora of complexes have been reported, for reviews see (Evans and Pickett, 2003; Bruschi et al., 2005; Kaur-Ghumaan and Stein, 2014; Simmons et al., 2014; Onoda and Hayashi, 2015; Schilter et al., 2016), most of which are homo- (FeFe) or hetero-bi-nuclear (NiFe, NiRu, and NiMn). The binuclear systems reported as electrocatalysts for the HER use mainly thiolates (with or without a pendant amine) and various electron-rich ligands (e.g., cyanides, phosphines, carbenes, *π*-accepting, and other non-innocent ligands) based on the structure known for the hydrogenase enzyme active sites (see Scheme 1). The binuclear systems reported hardly showed any activity under the conditions at which the enzyme functions and have limitations with respect to catalyst stability. The availability of an electron reservoir, balanced proton affinity, and a fine-tuning of the electronic and structural properties of a hydride were recently identified to be the most critical design aspects for bio-inspired HER catalysts (Sun et al., 2022).

Mono-nuclear catalysts, however, are less explored but have the advantage of an easier synthesis and a higher density of catalytic sites (see above). Those reported contain, among others, Fe (Kaur-Ghumaan et al., 2010; Natarajan et al., 2017), Co (Wilson et al., 2006; Dempsey et al., 2009; Artero and Saveant, 2014; Fang et al., 2014), and Ni (Wilson et al., 2006; Brazzolotto et al., 2016) (see Scheme 1).

Some of the coordinating ligands have been implicated to be directly or indirectly involved in the HER as electron-donors (Bigi et al., 2010), be partially redox-active (Straistari et al., 2017), or function as proton relays (DuBois, 2014).

Manganese is an exquisite catalyst for oxygen reduction/evolution (Hirahara et al., 2014; Wiechen et al., 2014). Like iron, manganese is easily available and abundant and shows a benign environmental profile compared to platinum. Mn-complexes reported until now have been mostly studied as catalysts for electro- or photo-catalytic organic oxidations/reductions (C–H hydroxylation; P450-type activity), alkene epoxidation, and CO_2_ reduction in homogenous or heterogeneous phases; for reviews see (Fernandez et al., 2022; Sorais, 2022). The complexes designed for these catalytic transformations use porphyrin and corrole derivatives or strong-chelating non-heme pyridylamino ligands, *π*-acceptors (CO), and pyridine-, diimine-, or N-heterocyclic carbene-based ligands.

However, the performance of manganese complexes in catalyzing HER has not received much attention until now, despite its abundance in the earth’s crust. The isoelectronic properties of non-native Mn^I^ with the bio-inorganic Fe^II^ suggest that Mn^I^-based complexes may also be suitable HER catalysts. However, there are very few Mn-based HER catalytic systems reported in the literature (Kaim et al., 2019; Kaim and Kaur-Ghumaan, 2019; Pan et al., 2019; Pan and Hu, 2020; Kaim and Kaur-Ghumaan, 2021; Yang et al., 2021) (see Scheme 1).

This prompted the design of a series of novel Mn^I^-based catalysts as an attractive alternative for hydrogen production. We, here, report the synthesis of a series of distorted octahedral mono-nuclear manganese (I) complexes which all show catalytic activity toward hydrogen production in an acidic solution. Their electrocatalytic proton reduction behavior and HER using TFA as a proton source are demonstrated. Carbon monoxide ligands are also found in the biological system (see above), phosphine ligands have electronic effects very similar to those of small inorganic CO and CN^−^ ligands, and sulfur coordination also occurs in the enzyme.

Upon variation of axial (PPh_2_(PhCOOH-*p*)) **1** and **3** vs. (PPh_3_) **2** and **4** and equatorial ligands containing heterocycles with only nitrogen **3** and **4** vs. one sulfur atoms **1** and **2,** their individual effects on the electronic structure and catalytic performance can be investigated. The electrochemical features of these complexes in the absence and presence of TFA are analyzed using cyclic voltammetry.

Redox transitions and catalytic mechanisms can be rationalized by quantum chemical calculations. The calculations show that complexes **1**–**4** display an inverted redox potential, whereas the nature of the axial ligand has almost no effect on catalytic performance, and the bidentate equatorial heterocycle is partially redox-active and serves as an initial site of protonation, following a first one-electron reduction event. An intramolecular proton transfer toward the central metal ion occurs after the second reduction event. Finally, an end-on H_2_-Mn(I) complex is formed from which the release of molecular hydrogen is facile and thermodynamically feasible.

## Materials and methods

Four mono-nuclear Mn-complexes were synthesized and structurally characterized using FTIR, ^1^H, and ^31^P NMR and MS spectrometry. For one complex, single crystals could be obtained with sufficient diffractions in order to be able to determine the crystal structure. The redox behavior of the four catalysts was investigated by cyclic voltammetry in the absence of acids to characterize the intrinsic redox processes for each catalyst. Finally, the catalytic behavior of the Mn-complexes toward the hydrogen evolution reaction (HER) was investigated in the presence of trifluoroacetic acid (TFA) in acetonitrile. The structural parameters, redox potentials, and catalytic behavior of all the four mono-nuclear Mn-complexes were obtained from quantum chemical calculations (using Density Functional Theory, DFT). These results are compared with the corresponding experimentally observed results in each section. The experimental and computational details are discussed in detail in the electronic supporting information (ESI).

### Synthesis and characterization of mononuclear Mn complexes

**SCHEME 2:**
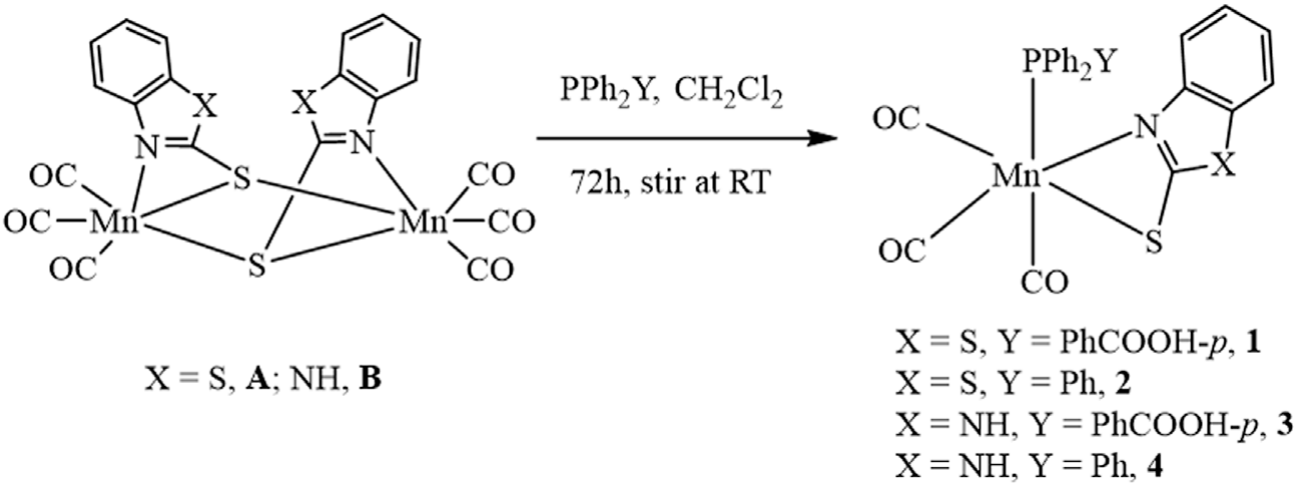
Scheme for synthesis of complexes **1–4**.

Mononuclear Mn complexes *fac*-[(Mn(CO)_3_(κ^2^-S_2_NC_7_H_4_)(PPh_2_(PhCOOH-*p*))] **1**, *fac*-[(Mn(CO)_3_(κ^2^-S_2_NC_7_H_4_)(PPh_3_)] **2** and *fac*-[Mn(CO)_3_(κ^2^-SN_2_C_7_H_5_)(PPh_2_(PhCOOH-*p*)] **3** were synthesized by the reaction of [(Mn(CO)_3_(µ-S_2_NC_7_H_4_))_2_] **A** and [(Mn(CO)_3_(µ-SN_2_C_7_H_5_)_2_)] **B** complexes with triphenylphosphine (**TL**_**1**_, PPh_3_) and 4-(diphenylphosphino)benzoic acid (**TL**_**2**_, PPh_2_(PhCOOH-*p*)) ligands in dichloromethane solvent for 72 h at room temperature as shown in Scheme 2. For comparison, the Mn analog *fac-*[(Mn(CO)_3_(*κ* ^2^-SN_2_C_7_H_5_)(PPh_3_)] **4** was also synthesized according to the published protocol (Scheme 2) (Abedin et al., 2018). All the complexes **1**–**4** were characterized using different spectroscopic techniques such as FTIR, ^1^H, and ^31^P NMR spectroscopy and MS spectrometry (see Supplementary Figures S1–S7, ESI).

### Computational details

Most computational details are given in the ESI. The GGA BP86 functional (Perdew, 1986; Becke, 1988) and the hybrid B3LYP functional (Vosko et al., 1980; Lee et al., 1988; Becke, 1993; Stephens et al., 1994) with all-electron def2-TZVP basis sets (Weigend and Ahlrichs, 2005) and dispersion corrections (Grimme et al., 2010) were used. All structures were characterized as minima by the absence of imaginary frequencies. In order to account for solvation effects, the implicit COSMO solvation model was used (Klamt and Schüürmann, 1993; Schäfer et al., 2000).

## Results and discussions

### Crystallization and structural characterization of Mn-complex 1

**FIGURE 1:**
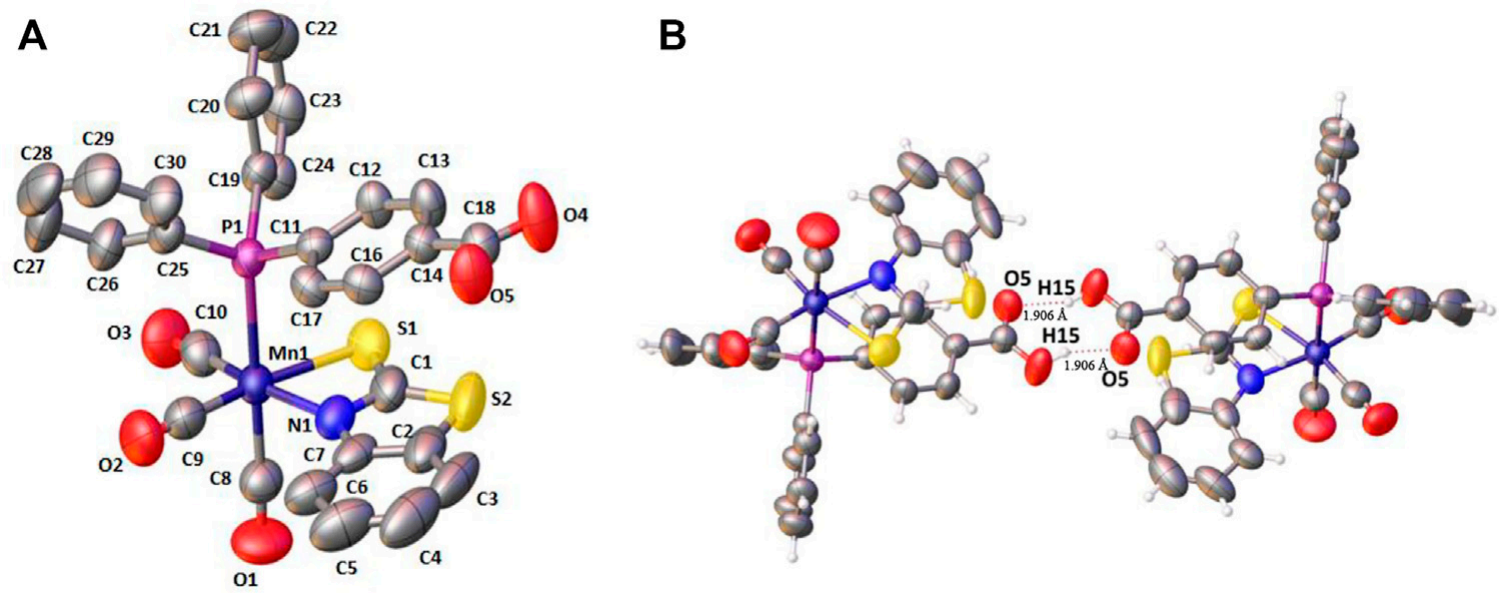
**(A)** X‒ray crystal structure for *fac-*[(Mn(CO)_3_(*κ* ^2^-S_2_NC_7_H_4_)(PPh_2_(PhCOOH-*p*))] **1**. Hydrogen atoms have been omitted for clarity. **(B)** Hydrogen bonding in the crystal structure of **1**.

For complex **1**, single-crystals could be obtained, which were analyzed by single-crystal X-ray diffraction. The crystallographic parameters and selected bond lengths and bond angles of complex **1** are reported in Supplementary Tables S1 and S2 in the supporting information (see ESI). Since the coordination sphere of complexes **2**–**4** is very similar, no significant structural differences are to be expected. X-ray structure analysis confirms the equatorial chelation by 2-mercaptobenzothiazole through N1 and S1; axial 4-(diphenylphosphino)benzoic acid and three carbonyl groups completed a distorted octahedral geometry (see Figure 1A). The N and S atoms of the chelating ligand and two carbonyl groups occupy the equatorial positions. In the crystal, **1** dimerizes by forming two intermolecular hydrogen bonds between the benzoic acid entities (Figure 1B). (Braga et al., 2001)

### Computational characterization of mono-nuclear Mn-complexes

Complexes **1**–**4** were structurally optimized using dispersion-corrected density functional theory (DFT). In the following section, only BP86 structural parameters are discussed throughout the manuscript, while B3LYP results are given in the ESI.

**FIGURE 2:**
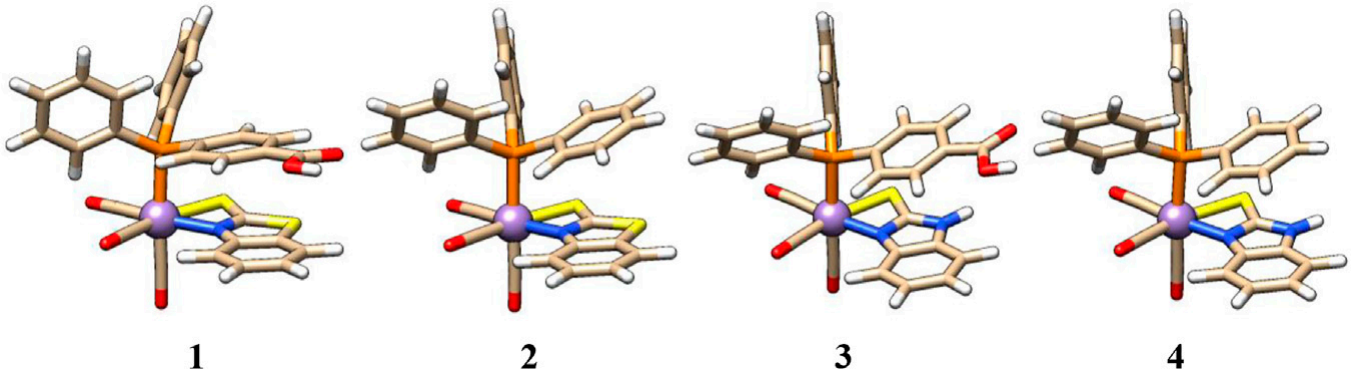
Structures of complexes **1–4** with identical first coordination spheres and variation of atomic composition in the second shell.

The structure of complex **2** is very similar to that of complex **1,** except for the axial PPh_3_ ligand that replaces the PPh_2_(PhCOOH-*p*) ligand. Similarly, in complexes **3** and **4**, Mn is coordinated with five ligands in a distorted octahedral geometry. The arrangement of axial PPh_2_Y and CO ligands in complexes **3** and **4** is the same as that in **1** and **2**, respectively, except for the equatorial chelating ligand. Here, a bidentate 2-mercaptobenzimidazole ligand is present in an equatorial position in complexes **3** and **4** (see Figure 2). The first coordination sphere is identical in all four complexes, as shown in Figure 2.

Neither equatorial Mn‒N bond distances (2.06, 2.06, 2.06, and 2.06 Å, in **1**–**4**) or Mn‒S bond distances (2.49, 2.49, 2.52, and 2.52 Å, in **1**–**4**), nor axial Mn‒P bond distances (2.37, 2.36, 2.36, and 2.36 Å, in **1**–**4**) are significantly influenced upon modifications of the equatorial and axial ligands. The mild distortion from octahedral symmetry can be seen in the angle < (CO_ax_-Mn-PR_3_) for **1**–**4** of 175°, 176°, 175°, and 175°; and the equatorial < (N‒Mn‒S) angle slightly deviating (<N‒Mn‒S **=** 64°-68°) from 90°. They, indeed, show that there are no large structural differences between complexes **1**–**4** (Supplementary Tables S3–S6).

### Characterization of redox activity

#### Mn-complex reduction in the absence of acid

The intrinsic redox behavior of complexes **1**–**4** was investigated by performing cyclic voltammetric (CV) experiments in CH_3_CN using 0.1 M [N(n-Bu_4_)][PF_6_] as the supporting electrolyte at a scan rate of 0.1 Vs^‒1^. The CVs of the complexes for oxidation are shown in Supplementary Figure S8 (see ESI). Scan rate linear dependence studies show that all the redox processes are diffusion-controlled (see Supplementary Figure S8, ESI).

**FIGURE 3:**
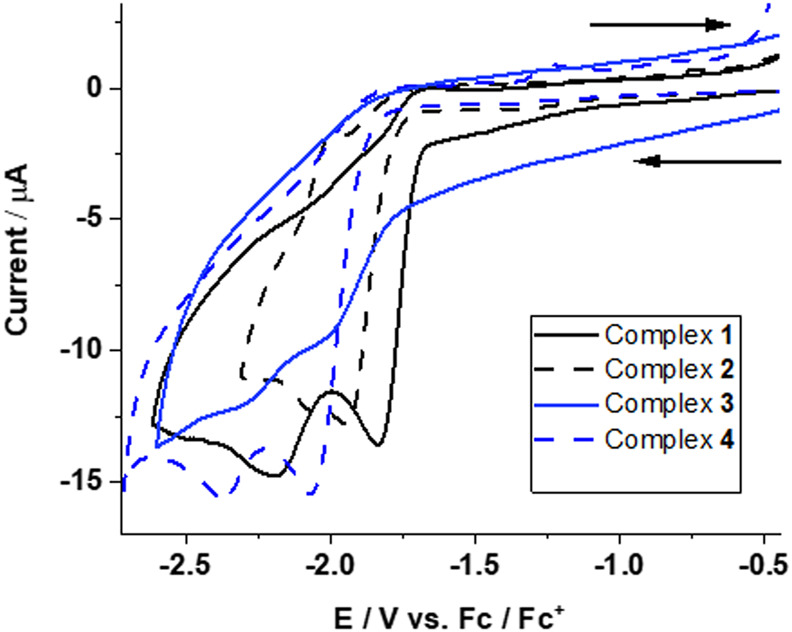
Cyclic voltammograms for reduction of complexes **1**–**4** in CH_3_CN at a scan rate of 0.1 V s^−1^. Assignment of redox transitions can be done by quantum chemical calculations (see text for details).

Complexes **1** and **3** (with PPh_2_(PhCOOH-*p*) ligand) each show two irreversible reduction peaks at $E_{\mathrm{pc}}^{1}$ = ‒1.83 (**1**), ‒1.99 (**3**) V and *E* ^2^_pc_ = ‒2.19 (**1**), ‒2.30 (**3**) V (Figure 3).

Complexes **2** and **4** (with PPh_3_ ligand) also exhibited two irreversible reduction peaks at $E_{\mathrm{pc}}^{1}$ = ‒1.93, ‒2.06 and *E* ^2^_pc_ = ‒2.07, ‒2.36 (Figure 3) vs. Fc/Fc^+^, respectively. This clearly indicates only minor control of redox transitions by equatorial and axial coordination. The irreversibility of the redox transitions clearly indicates large and irreversible structural re-arrangements.

The structural interpretation of CV is difficult and can only be indirect. Quantum chemically calculated redox potentials allow the assignment of redox transitions to structural changes when calculated potentials and experiments agree to within a ∼100 mV. In order to assign redox CV peak positions, redox potentials for the sequential two-one electron reduction steps (Eqs 1, 2) plus one two-electron reduction step were calculated (Eq. (3)) (Supplementary Table S7, ESI).

$$1+e^{-}\to \;1^{-}$$ (1)

$$1^{-}+e^{-}\to \;1^{2-}$$ (2)

$$1+2e^{-}\to \;1^{2-}$$ (3)

The reduction potentials of complexes **1**–**4** are very close and occur at ‒1.63, ‒1.67, ‒1.62, and ‒1.65 V for the first and at ‒2.16, ‒2.18, ‒2.20, and ‒2.19 V for the second one-electron reduction, respectively. This shows that the electronic structure of all four complexes is very similar.

The two-electron reduction for complexes **1**–**4**, however, is suggested to occur at potentials of ‒1.89, ‒1.92, ‒1.91, and ‒1.92 V and are thus at less negative potentials than the second one-electron reduction process (inverted redox potential).

Experimentally, the first one-electron reduction near ‒1.6 V for complexes **1**–**4** could not be resolved, which was also observed for mono-nuclear iron complexes (Natarajan et al., 2017).

The DFT calculated two-electron reduction potentials, as well as the second one-electron reduction potentials, are in excellent agreement with the experimentally observed peaks at ‒1.83, ‒1.93, ‒1.99, and ‒2.06 V and ‒2.19, ‒ 2.07, ‒2.30, and ‒2.36 V, respectively. This allows a definite assignment of observed reduction peaks in the CVs of complexes **1**–**4** corresponding to the formation of doubly reduced species (**1** ^**2‒**^ - **4** ^**2‒**^), whereas the second CV peak is the transition **1** ^**-**^/**1** ^**2-**^ to **4** ^**-**^/**4** ^**2-**^.

It can be suggested that the mono-reduced species **1** ^**-**^/**2** ^**-**^/**3** ^**-**^/**4** ^**-**^ are too short-lived to be resolved in the CV. Given the facile two-electron reduction process, it is likely that the intermediates **1** ^2-^/**2** ^2-^/**3** ^2-^/**4** ^2-^ are the first stable intermediates. This agrees with the suggested reaction mechanism (see below) and an EECC sequence of electrochemical (E) and chemical (C) events.

#### Computed structural changes during redox transitions

Quantum chemical calculations are able to elucidate structural changes in the electronic structure of transition metal complexes during redox transitions. Such detailed information is difficult to obtain experimentally. Upon the first one-electron reduction, the Mn−S bond in complexes **1** ^**‒**^ **–4** ^**‒**^ dissociates and opens a free coordination site at the Mn center and the ligand sulfur atoms. This breaking of the Mn−S bond can explain the irreversible character of the peaks in the CVs. The calculated Mn‒S distances are elongated (by 0.7–1.0 Å) to 3.24, 3.30, 3.44, and 3.51 Å in **1** ^**‒**^, **2** ^**‒**^, **3** ^**‒**^, and **4** ^**‒**^, respectively.

**FIGURE 4:**
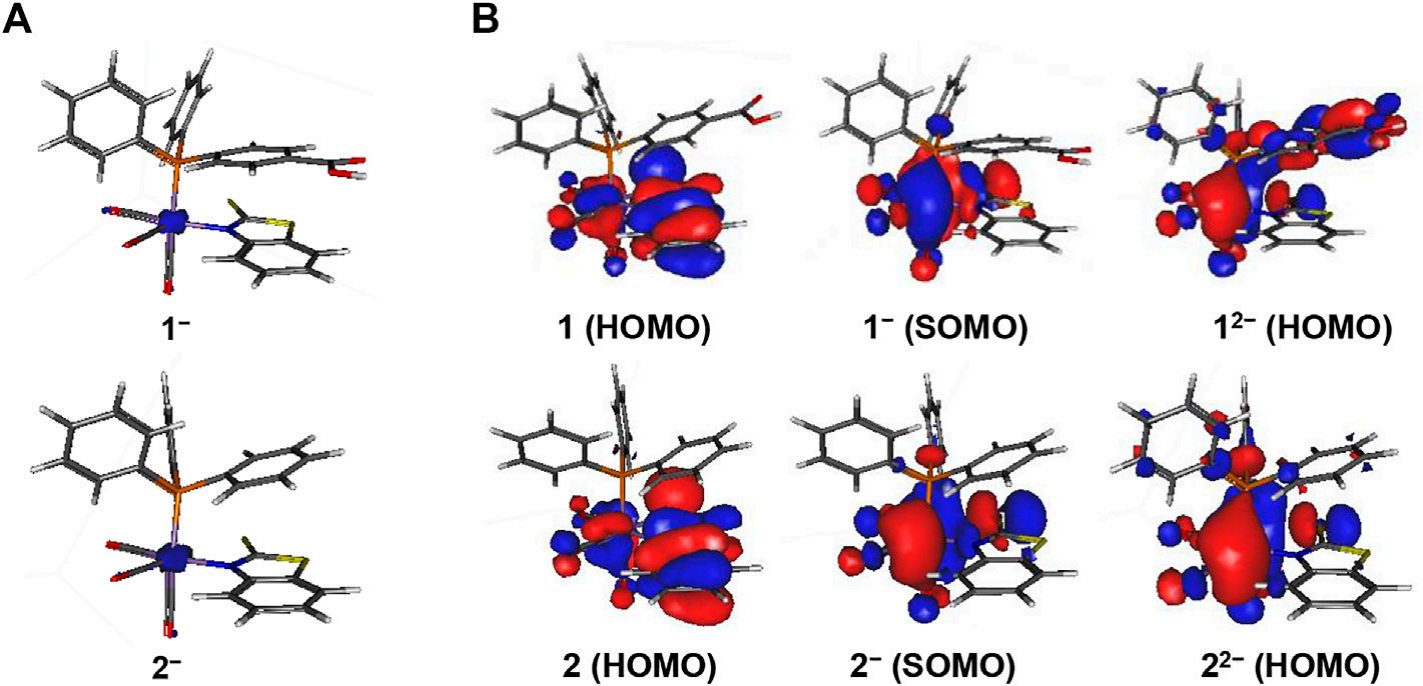
Display of **(A)** unpaired spin density distribution (at an isocontour value = 0.01e) and **(B)** highest occupied molecular orbitals of complexes **1** and **2** (at an isocontour value = 0.02e). The PhCOOH-*p* group in **1** affords a partial ligand-based second reduction event in **1** ^**-**^/**1** ^**2-**^ whereas **2** ^**-**^/**2** ^**2-**^ is metal-centered.

No further structural changes are observed in the reduced complexes **1** ^**‒**^ **– 4** ^**‒**^. The calculated unpaired spin density is localized mainly on the Mn-atom (Figure 4 and Supplementary Figure S9) in the reduced species, which confirms that the first one-electron reduction event is primarily metal-centered. The close redox potentials for **1–4** show that minor changes in the second coordination sphere do not affect the reduction potential. From an analysis of the molecular orbitals HOMO and SOMO, it can be seen that the first one-electron reduction in **1–4** is mainly Mn-based and only partially involves the S-atom of the chelating ligand (Figure 4 and Supplementary Figure S9).

Addition of a second electron leads to the doubly reduced species **1** ^**2‒**^, **2** ^**2‒**^, **3** ^**2‒**^, and **4** ^**2‒**^ which are characterized by a further increase in Mn‒S bond lengths (by 0.2–0.4 Å) to 3.55, 3.66, 3.67, and 3.79 Å. Changes of other Mn‒ligand bond distances are negligible (see Supplementary Tables S3–S6, ESI). The structures of **1** ^**2−**^–**4** ^**2−**^ are distorted due to re-orientations of the axial ligands. The < (P‒Mn‒CO_axial_) angle deviates significantly from the ideal 180° (148°, 136°, 146°, and 127° in **1** ^**2−**^–**4** ^**2−**^ complexes). The second one-electron reduction is facile but less than the first one-electron step (by 49–57 kJ/mol).

The reduction **1** ^**-**^/**1** ^**2-**^ and **3** ^**-**^/**3** ^**2-**^ is partially ligand-based due to the delocalization of electrons over the PhCOOH-*p* group, giving stable resonating structures of the reduced ligand. For **2** ^**-**^/**2** ^**2-**^ and **4** ^**-**^/**4** ^**2-**^, however, the second reduction is mainly metal-centered (Figure 4). The second reduction does not have any significant effect on the reduction potential of the four complexes.

#### Catalytic activity of mono-nuclear manganese complexes

**FIGURE 5:**
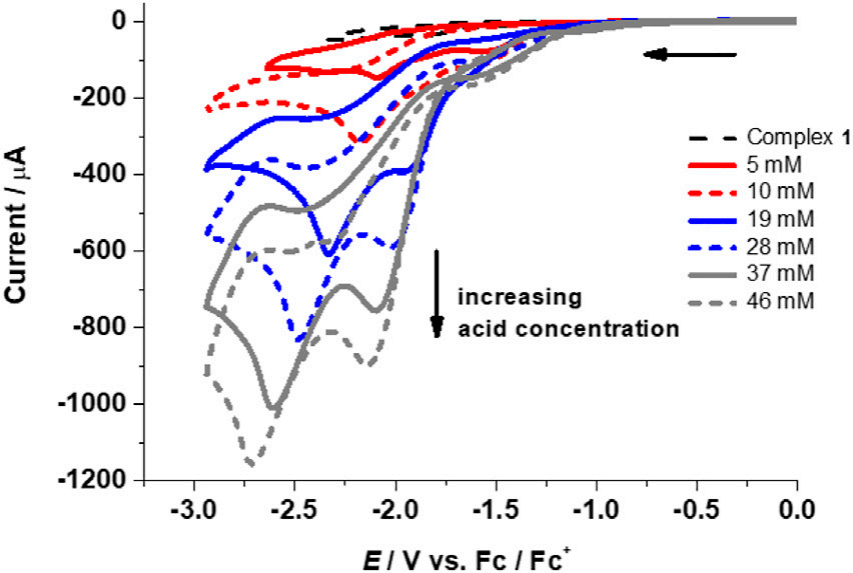
CVs for complex **1** (1 mM) in the absence (top curve, dashed black line) and presence of TFA (5–46 mM) at 0.1 Vs^‒1^.

The performance of complexes **1**–**4** as HER catalysts in the presence of trifluoroacetic acid (TFA) was explored in acetonitrile (p*K*_a_ of TFA in CH_3_CN = 12.7, *E* ^0^ in CH_3_CN = ‒ 0.89 V). Addition of TFA to **1**-**4** results in two irreversible waves with increasing peak intensity upon further addition of acid, thus indicating catalytic electrochemical proton reduction (Figure 5; Table 1 and Supplementary Figure S10, ESI). In the absence of a catalyst, negligible potential and currents were observed between ‒1.5 and ‒2.6 V for proton reduction in the presence of TFA in CH_3_CN at 0.1 Vs^‒1^ (Supplementary Figure S11, see ESI).

**TABLE 1:** Electrochemical data for complexes **1–4** in acetonitrile.

Complex	*E*_pc_^red^/V	${\left(\frac{i_{cat}}{i_{p}}\right)}_{\max}$	*TOF*/s^−1^	*E*_cat_/V	Overpotential/V	Applied potential during bulk electrolysis (Time of electrolysis/min)	TON
1	−1.83, −2.19	64	805	−1.87	0.96	‒2.0 (30)	0.19
2	−1.93, −2.07	48	445	−1.88	0.99	‒2.2 (30)	0.44
3	−1.99, −2.30	42	340	−1.88	0.99	‒2.2 (30)	0.58
4	−2.06, −2.36	68	890	−1.95	1.06	‒2.2 (30)	0.71

Initial addition of acid shows reductions at or slightly more negative reduction potentials than the complex peaks and/or followed by the appearance of new peaks on the addition of ∼15–20 mM of acid. This suggests a possible reduction of the complexes followed by protonation with the addition of acid. The peaks shift toward more negative reduction potential with increasing acid concentration and level off at ∼189 mM (**1**), ∼123 mM (**2**), ∼100 mM (**3**), and ∼143 mM (**4**) of TFA. This shows that the increase in acid addition leads to a sustained reduction. The levelling off indicates that most of the reactive species (at the electrode/electrolyte interface) have already been oxidized or reduced.

The first peak can be assigned to be the catalytic wave with a half wave potential *E*_cat_ of ‒1.87 V (**1**), ‒1.88 (**2**), ‒1.88 (**3**), and ‒1.95 (**4**) (Figure 5; Table 1 and Supplementary Figure S10, ESI). The catalytic wave corresponding to the electrocatalytic reduction of protons to hydrogen was further confirmed by bulk electrolysis experiments in acetonitrile for each complex **1–4** (0.25 mM) between ‒2.0 to −2.25 V in CH_3_CN/0.1 M [N(n-Bu_4_)][PF_6_]/6 mM TFA (Supplementary Figure S12, ESI). From the controlled potential electrolysis (CPE) experiments for the complexes, TONs were calculated to be 0.19 (**1**), 0.44 (**2**), 0.58 (**3**), and 0.71 (**4**) (Fu et al., 2015a; b). Evaluation of CVs measured at different scan rates for different TFA concentrations indicates the dependence of *i*_cat_ on the scan rate (Supplementary Figure S13, ESI). From the linear plot of *i*_cat_ vs. [catalyst], it is evident that the reaction is first order in complex concentration for **1–4** at fixed acid concentrations (Supplementary Figure S14, ESI). The overpotential for complexes **1–4** was calculated using Evans’s method to be in the range of 0.96–1.06 V (using *E*_cat/2_) (Felton et al., 2006; Helm et al., 2011).

The appearance of a second reduction peak in the CVs of **1–4** in the presence of TFA might originate from the formation of stable adducts between the acid (AH) and its conjugate base (A^−^). Homoconjugation complicates the determinations of the thermodynamics of H_2_ evolution since AH and AHA^−^ may act as proton sources for hydrogen production. Homoconjugation in acetonitrile has been reported to occur at high concentrations of weak acids and influence the acid**–**base equilibrium above the association constant *K*_c_. Trifluoroacetic acid (TFA) displays a high value of *K*_*c*_ = 103.9 and is therefore expected to undergo homoconjugation to a significant extent even at low concentrations. This process affects the p*K*_a_ of the acid in CH_3_CN, leading to different *E* ^0^ values for the acid (which can be calculated using Eq. 4), which can, thus, be challenging in determining accurate overpotentials (Appel and Helm, 2014).

$$E_{\mathrm{AH/H2, A}}^{0}{\;}^{‐}=E_{\mathrm{H +/H2}}^{0}-\text{2.3RT/F}\times \text{p}K_{\text{a}}$$ (1)

**FIGURE 6:**
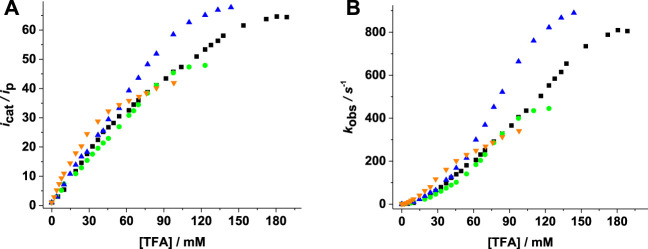
**(A)** $\frac{i_{cat}}{i_{p}}$ (first catalytic peak) vs [acid]; **(B)** dependence of *TOF* (s^−1^) on [acid] for complexes **1** (■), **2** (●), **3** (▼), and **4** (▲) (1.5 mM) in 0.1 M [N(n−Bu_4_)][PF_6_]/CH_3_CN.

Figure 6 shows the increase of catalytic current and *TOF* with acid concentration. Higher $\frac{i_{cat}}{i_{p}}$ and *TOF* values for complex **4** are slightly larger than the values for complexes **1**–**3**, indicating faster turnover of complex **4**.

The Mn complexes **1**–**4** showed activity with lower *TOF*/TON values than previously reported Mn complexes but improved $\frac{i_{cat}}{i_{p}}$ values at comparable overpotentials (Kaim et al., 2019; Kaim and Kaur-Ghumaan, 2019; Pan et al., 2019; Pan and Hu, 2020; Yang et al., 2021). The mononuclear Mn complex with heterocyclic (N,S; S,S) and phosphaadamantane ligands (Kaim and Kaur-Ghumaan, 2021) showed activity in both organic and partially aqueous media. The Mn complexes reported in this article open the possibility of further exploring electrocatalysis in aqueous media and also the path of immobilizing the catalyst onto electrode surfaces via the -COOH substituted phosphine ligand.

#### Mechanism of hydrogen evolution reaction

**SCHEME 3:**
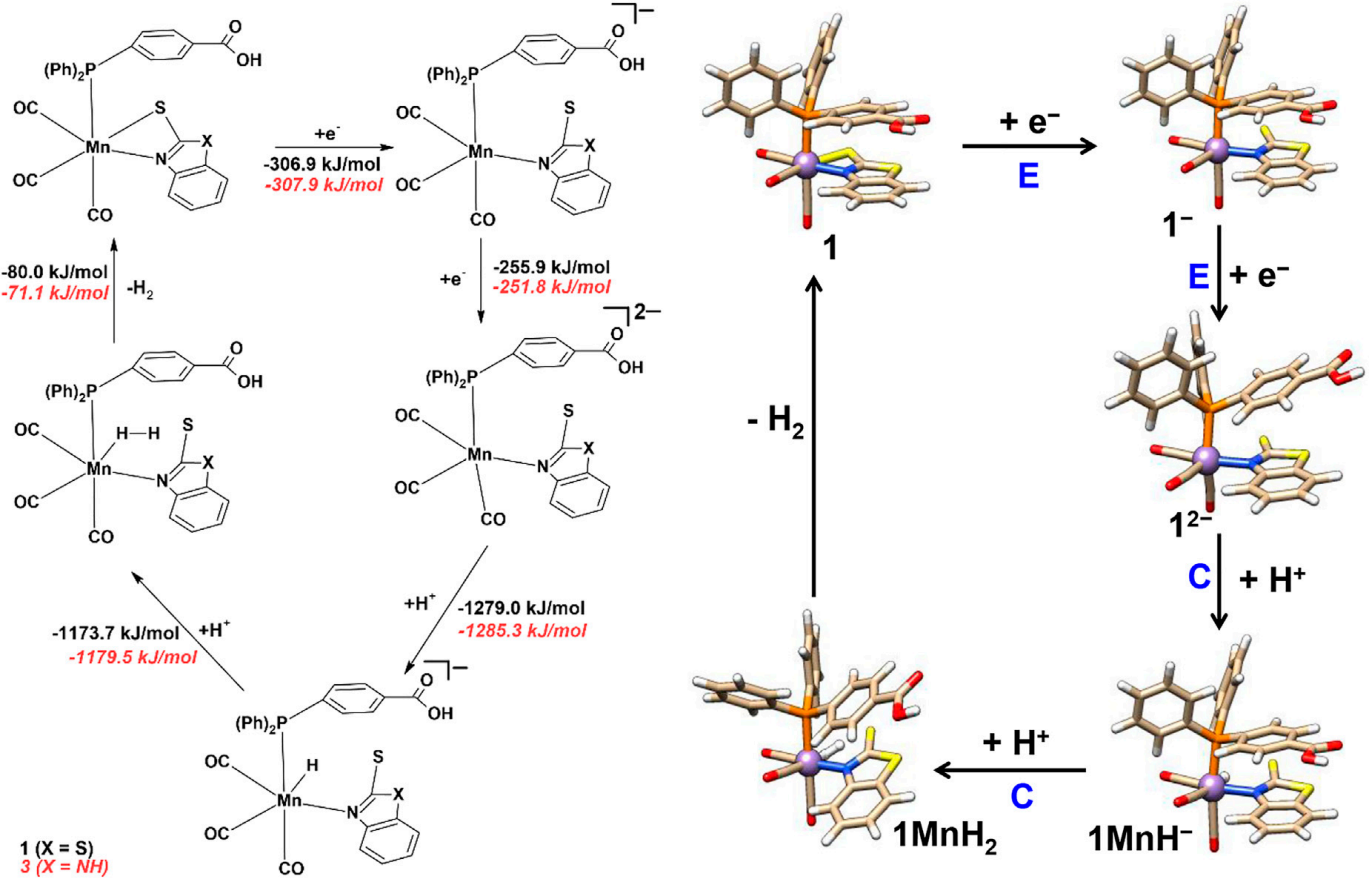
Proton reduction and hydrogen evolution mechanism of complexes **1** and **3**.

**SCHEME 4:**
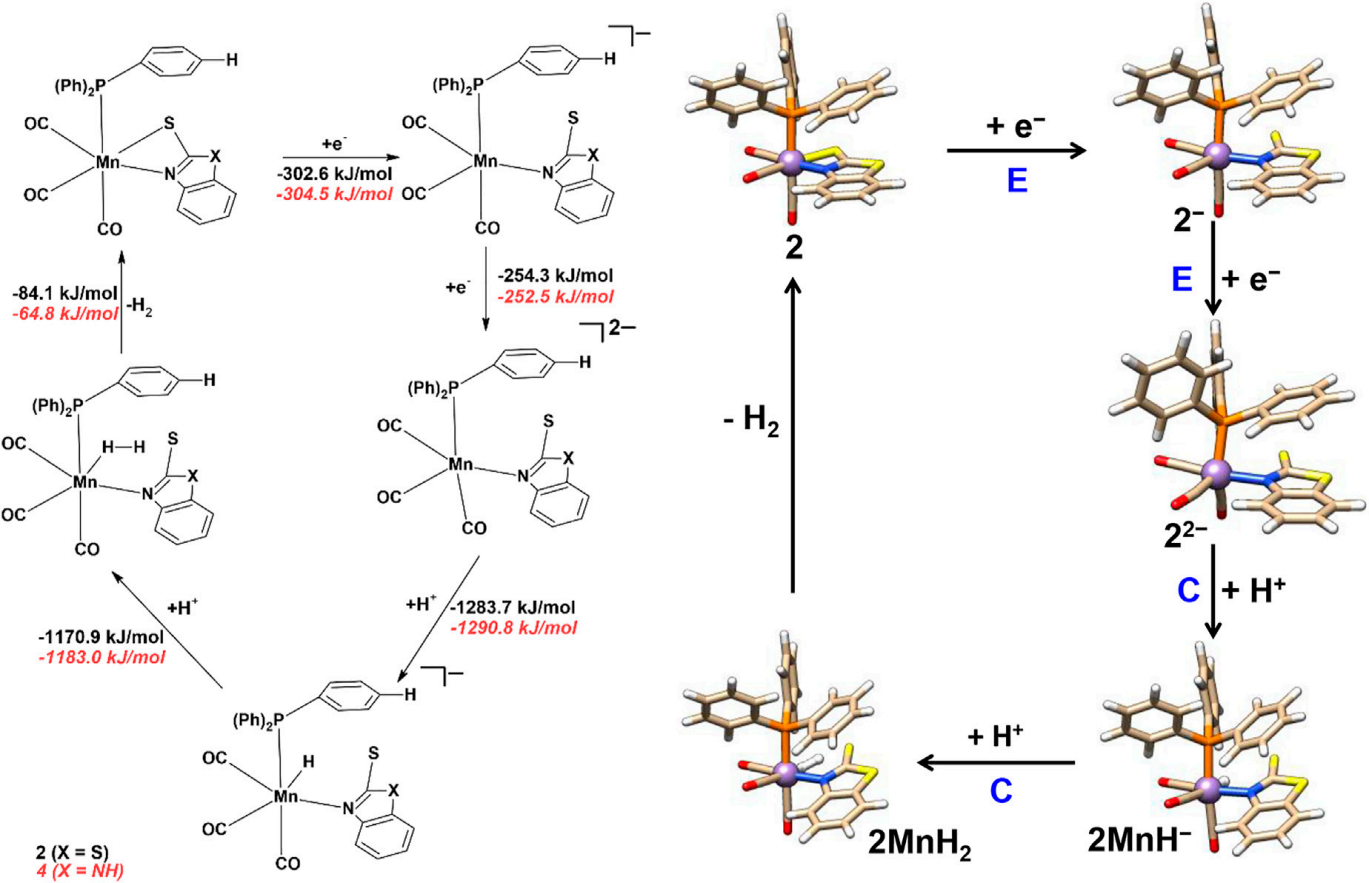
Proton reduction and hydrogen evolution mechanism of complexes **2** and **4**.

The electrochemical proton reduction can occur via ECEC or EECC (E = electrochemical and C = chemical step) mechanisms for all the four mononuclear Mn complexes. Changes in Gibbs free energies (ΔG) for each reaction step are reported in Schemes 3 and 4, while the results in the absence of solvent are reported in the ESI (Supplementary Tables S8–S11, ESI).

#### ECEC vs. EECC mechanistic pathways

After one-electron reduction (E), the Mn‒S bond dissociates to give **1** ^**‒**^ and **3** ^**‒**^ species (see above) and provides an accessible site for protonation at either Mn or S atoms (see Scheme 3).

In the ECEC pathway, protonation (C) at the sulfur atom is energetically favorable (ΔG of ‒1,148.1 and ‒1,153.6 kJ/mol) to form **1SH** and **3SH** species with S‒H bond distances of 1.45 and 1.44 Å, respectively. Direct protonation of Mn was not feasible, and the proton reverts to the sulfur again. Only after the second one-electron reduction step (E), an intramolecular proton transfer occurs spontaneously from the S atom to the Mn atom to form **1MnH** ^**−**^ and **3MnH** ^**−**^. The Mn‒H bond lengths are 1.58 Å in **1MnH** ^**−**^ and **3MnH** ^**−**^. The formation of **1MnH** ^**−**^ and **3MnH** ^**−**^ is energetically feasible with ΔG of ‒386.8 and ‒383.4 kJ/mol.

The second protonation step for **1MnH** ^**−**^ and **3MnH** ^**−**^ occurs at the manganese hydride to give end-on H_2_ complexes **1MnH**_**2**_ and **3MnH**_**2**_ (ΔG = ‒1,173.7 and ‒1,179.5 kJ/mol) with H‒H bond distances of 0.93 Å and 0.89 Å, respectively. The binding of H_2_ to manganese is end-on with different Mn‒H distances of 1.66, 2.05 Å in **1MnH**_**2**_ and 1.68, 1.99 Å in **3MnH**_**2**_. The second hydrogen atom only weakly interacts with the S (S-H = 1.76–1.99 Å).

The ECEC reaction mechanism is devoid of any significant structural changes, and the complex remains intact except for a Mn‒S bond cleavage in the first electron reduction step (see Supplementary Tables S3–S5). Finally, upon H_2_ release from **1MnH**_**2**_ and **3MnH**_**2**_, the Mn‒S bond re-forms to regenerate complexes **1** and **3**. The release of molecular hydrogen is an exergonic process with ΔG values of ‒80.0 and ‒71.1 kJ/mol (Supplementary Scheme S1, see ESI).

In the EECC mechanism (Scheme 3), upon the addition of the second electron, the axial aromatic ligand (see above) significantly deviates from the ideal 180°, and the equatorial Mn‒S bond length increases by 0.2–0.3 Å in species **1** ^**2‒**^ and **3** ^**2‒**^. The remaining other Mn‒ligand bond distances remained almost unchanged. In **1** ^**2‒**^ and **3** ^**2‒**^ possible sites for protonation are available at both the Mn and S atoms, and the first protonation is found to occur at Mn to form **1MnH** ^**−**^ and **3MnH** ^**−**^ (a similar structure is observed in the ECEC mechanism, see above). The ΔG values of protonation to obtain **1MnH** ^**−**^ and **3MnH** ^**−**^ are ‒1,279.0 and ‒1,285.3 kJ/mol, respectively, and thus almost indistinguishable. The optimized structural parameters of all the reaction intermediates are listed in Supplementary Tables S3–S5 (ESI). The remaining steps of hydrogen generation and release in the EECC mechanism are the same (see Scheme 3) as discussed above for the ECEC mechanism.

Among the two possible hydrogen releasing mechanisms, the two-electron reduction of the catalyst followed by two protonation events, i.e., the EECC mechanism appears to be more plausible to occur in the presence of TFA (p*K*_a_ of TFA in CH_3_CN = 12.7) since the mono-reduced species could not be observed in the CV. Furthermore, the di-anion more easily reduces the proton (by ∼131 kJ/mol) in the EECC mechanism than that of the mono-anion in the ECEC mechanism. Nevertheless, the use of a very strong acid (with p*K*_a_ < 6–8) could favor the ECEC mechanism for the HER.

Complexes **2** and **4** follow the same EECC and ECEC mechanism as discussed above for complexes **1** and **3** and are thus not discussed in detail here. Here, we briefly discuss the EECC mechanism for complexes **2** and **4**. The ECEC proton reduction mechanism and corresponding Gibbs free energies of complexes **2** and **4** are given in ESI, Supplementary Scheme S1. The optimized structural parameters of all the reaction intermediates are listed in Supplementary Tables S4–S6.

In the EECC mechanism (Scheme 4) of complexes **2** and **4**, the protonation (C) occurs at the Mn atom of **2** ^**2‒**^ and **4** ^**2‒**^ (Mn‒H = 1.58 Å) to form **2MnH** ^**−**^ and **4MnH** ^**−**^ species. In the next step (C), the second proton binds to the terminal hydride to form **2MnH**_**2**_ and **4MnH**_**2**_ species with the H‒H distance of 0.97 Å and 0.95 Å, respectively. The Mn‒H distances are 1.65, 2.13 Å in **2MnH**_**2**_ and 1.66, 2.14 Å in **4MnH**_**2**_, which indicates an end-on metal-H_2_ complex. In the final step, the **2MnH**_**2**_ and **4MnH**_**2**_ complexes release molecular hydrogen with ΔG of ‒84.1 and ‒64.8 kJ/mol and regenerate the complexes **2** and **4**.

It is worth mentioning that the presence of different ligands in complexes **1**–**4** causes only a minimal change in the structures as well as in the ΔG value (by 0.4–19 kJ/mol) of the electrochemical (E) and chemical (C) steps of the HER of these Mn-complexes**.**

## Conclusion

Mononuclear Mn complexes **1**–**4** are able to catalyze the electrochemical proton reduction and the HER with TFA as a proton source. After careful characterization with NMR, FT-IR, UV–Vis spectroscopy, cyclic voltammetry, and quantum chemical calculations, we were able to assign and interpret the redox transitions and suggest mechanistic pathways for hydrogen generation.

The short-lived mono-reduced species were shown not to be part of the catalytic cycle. Only the two-electron reduced species (**1**–**4**)^2**‒**^ at −1.9 V and the second one-electron transition (**1**–**4**)^1**‒**^/(**1**–**4**)^2**‒**^ at **−**2.2 V can be observed. This implies a hydrogen evolution mechanism starting from the two-electron reduced intermediate along with two sequential protonation events at the Mn atom and then the formation of a loosely coordinated end-on H_2_ complex.

Variation of ligands in the second coordination sphere affects the redox potentials, reaction mechanism, and catalytic efficiency only to a minor degree.

The high cost of platinum catalysts used in hydrogen fuel cells is a limiting factor in the commercialization of fuel cell electric vehicles in addition to its negative environmental impact. The current global demand for platinum (approximately 200 t p. a.) is high compared to the available platinum reserves, and only about 17% of the used platinum is recycled (Duclos et al., 2017).

The quest for alternative, more cost-effective catalysts to maintain the efficiency of hydrogen fuel cells is ongoing.

With the introduction of manganese as an additional earth-abundant metal atom into the series of mono-nuclear hydrogen generating catalysts (in addition to Fe, Co, Ni, Ru), the synthetically accessible chemical space opens up. Its low cost and abundant availability, plus a benign environmental profile, make it an interesting candidate for the HER.

Approaches to control catalytic performance and catalyst stability by intra-molecular non-covalent (Natarajan et al., 2017) (Natarajan et al., 2022) and covalent (Pullen et al., 2019) interactions or the reaction mechanism by introducing additional sites of protonation (Wilson et al., 2006; Liu et al., 2013; Pandey et al., 2021) or modifications of the first coordination shell ligands will allow the design of more efficient catalyst candidates for sustainable hydrogen production.

## Data Availability

The datasets presented in this study can be found in online repositories. The names of the repository/repositories and accession number(s) can be found in the article/Supplementary Material. CCDC entry 2189879 **(1)** contains the crystallographic data for this paper. These data can be obtained free of charge from the Cambridge Crystallographic Data Centre (www.ccdc.cam.ac.uk/data_request/cif).
