# Supplementary material for: Mononuclear manganese complexes as hydrogen evolving catalysts
Source: Front Chem. 2022 Oct 7;10:993085. doi: 10.3389/fchem.2022.993085 (PMC9585328; doi:10.3389/fchem.2022.993085)
Supplement: Supplementary file 1 [file DataSheet2.pdf]

Supporting Information

Mononuclear Manganese Complexes as Hydrogen Evolving Catalysts

Vishakha Kaim[a], Meenakshi Joshi[b], Matthias Stein*,[b], Sandeep Kaur-Ghumaan*,[a]

[a]Department of Chemistry, University of Delhi, Delhi-110007, India

*Dr Sandeep Kaur-Ghumaan, sandeepkaur.du@gmail.com

[b]Max-Planck-Institute for Dynamics of Complex Technical Systems, Molecular Simulations and Design Group, Sandtorstrasse 1, 39106 Magdeburg, Germany

* Dr Matthias Stein, matthias.stein@mpi-magdeburg.mpg.de

Spectroscopic characterization

The ^{1}H NMR spectra of complexes **1** and **2** display resonance peaks in the range of 7.14-7.39 ppm. These peaks are associated with the aromatic protons of the ligands attached to the Mn atom. The $^{31}P\{^{1}H\}$ NMR spectra display singlet at 57.96 (**1**), 56.77 (**2**) and 50.61 (**3**) ppm for the coordinated phosphine ligands (**Figures S1 and S2**). The FTIR spectra of complexes **1-4** recorded in dichloromethane show absorption bands between 2021-1902 cm^{-1} (**Figure S3**), indicating that all carbonyl ligands in the complexes are terminal in nature.[1] With DFT also the absorption bands corresponding to the CO ligands are observed in the range of 2019-1920 cm^{-1} for complexes **1-4**. Complexes **1**, **2** and **3** exhibited molecular masses m/z at 611.98 $(\mathbf{1}+H)^{+}$, 568.00 ($\mathbf{2}^{+}$) and 595.02 $(\mathbf{3}+H)^{+}$, respectively, corresponding to calculated molecular weight for $(\mathbf{1}+H)^{+}$, 612.53; $\mathbf{2}^{+}$, 567.52, and $(\mathbf{3}+H)^{+}$, 595.48 respectively (**Figures S4-S6**). The UV-vis measurements for complexes **1-4** recorded in CH_3CN showed absorption bands in the range 300–330 nm, which could be assigned to dπ-dπ* transitions (**Figure S7**).

Experimental section

Materials and Physical Measurements

All solvents and chemicals were purchased from Sigma Aldrich and were used without further purification. The synthesis of complex **4** was carried out according to the procedure described in literature. JEOL 400 MHz NMR Spectrometer was used to record the ^{1}H and ^{31}P NMR at room temperature in $CDCl_3$ solution. Perkin Elmer FTIR Spectrometer was used for recording the FTIR spectra from CH_2Cl_2 solutions of the samples over the range 400–4000 cm^{-1}. The UV-vis spectra for complexes were recorded in CH_3CN on a Perkin-Elmer Lambda-spectrophotometer. Mass spectra were recorded with a Quadrupole Time-of-flight mass spectrometer with ESI and APCI source (Agilent G6530AA (LC-HRMS-Q-TOF)). Elemental (C, H, and N) analyses were performed on a Leco TruSpec Micro CHNS analyzer.

Synthesis of fac-[(Mn(CO)$_3$(κ^2 -S$_2$NC$_7$H$_4$)(PPh$_2$(PhCOOH-*p*))] 1

A dichloromethane solution (30 ml) of $[(Mn(CO)_3(\mu\text{-}S_2NC_7H_4))_2]$ (**A**) (100 mg, 0.16 mmol) and 4-(Diphenylphosphino)benzoic acid (**TL$_2$**) (147.16 mg, 0.32 mmol) was stirred at RT for 72 h. The solvent was then removed under reduced pressure and the crude product was chromatographed on a silica gel column. Elution with hexane/ethanol (9:1, v/v) resulted in two bands. A faster moving band gave traces of complex **A** whereas the slower moving band gave a yellow-coloured product as complex **1**.

Spectral data for 1:

Yield: 90 mg (90%). FTIR (ν_{CO}, CH_2Cl_2): 2018 (br), 1940 (br), 1902 (br) cm^{-1}. ^1H NMR (400 MHz, $CDCl_3$, 298 K, TMS): δ 7.44–7.31 (14H, aromatic protons, TL_2), 7.14 (m, 4H, $S_2NC_7H_4$) ppm. ^{31}P NMR (161.8 MHz, $CDCl_3$, 298 K): 57.96 ppm. ESI-MS (CH_3CN): 611.98, calcd: 611.53 $[M+H]^+$. Anal. calc. for $C_{29}H_{19}MnNS_2O_5P$ (%): C = 56.90, H = 3.10, N = 2.28, S = 10.46; found C = 56.29, H = 3.30, N = 2.68, S = 11.00.

Synthesis of fac-[(Mn(CO)$_3$(κ^2 -S$_2$NC$_7$H$_4$)(PPh$_3$)] 2

A dichloromethane solution (30 ml) of $[(Mn(CO)_3(\mu\text{-}S_2NC_7H_4))_2]$ (**A**) (100 mg, 0.16 mmol) and triphenyphosphine (**TL$_1$**) (88 mg, 0.32 mmol) was stirred at RT for 72h. The solvent was then removed under reduced pressure and the crude product was chromatographed on a silica gel column. Elution with hexane/ethanol (9:1, v/v) resulted in two bands. A faster moving band gave the traces of complex **A** whereas the slower moving band gave a yellow-coloured product as complex **2**.

Spectral data for 2:

Yield: 59 mg (68%). FTIR (ν_{CO}, CH_2Cl_2): 2017 (br), 1937 (br), 1902 (br) cm^{-1}. ^1H NMR (400 MHz, $CDCl_3$, 298 K, TMS): δ 7.39–7.35 (15H, aromatic protons, TL_1), 7.21 (m, 4H, $S_2NC_7H_4$) ppm. ^{31}P NMR (161.8 MHz, $CDCl_3$, 298 K): 56.77 ppm. ESI-MS (CH_3CN): 568.00, calcd: 567.52 $[M]^+$. Anal. calc. for $C_{28}H_{19}MnNS_2O_3P$ (%): C = 59.20, H = 3.34, N = 2.46, S = 11.27; found C = 59.96, H = 3.56, N = 2.53, S = 11.23.

Synthesis of fac-[(Mn(CO)$_3$(κ^2 -SN$_2$C$_7$H$_5$)(PPh$_2$(PhCOOH-*p*))] 3

A dichloromethane solution (30 ml) of $[(Mn(CO)_3(\mu\text{-}SN_2C_7H_5))_2]$ (**B**) (100 mg, 0.16 mmol) and 4-(Diphenylphosphino)benzoic acid (**TL$_2$**) (88 mg, 0.32 mmol) was stirred at RT for 72h. The solvent was then removed under reduced pressure and the crude product was chromatographed on a silica gel column. Elution with hexane/ethanol (9:1, v/v) resulted in two bands. A slower moving band gave a yellow-coloured product as complex **3**.

Spectral data for 3:

Yield: 48 mg (66%). FTIR (ν_{CO}, CH_2Cl_2): 2021 (br), 1938 (br), 1905 (br) cm^{-1}. ^1H NMR (400 MHz, $CDCl_3$, 298 K, TMS): δ 7.42–7.19 (18H, aromatic protons), 7.68 (NH proton) ppm. ^{31}P NMR (161.8 MHz, $CDCl_3$, 298 K): 50.61 ppm. ESI-MS (CH_3CN): 595.02, calcd: 595.48 $[M+H]^+$. Anal. calc. for $C_{29}H_{20}MnN_2SO_5P$ (%): C = 58.53, H = 3.36, N = 4.70, S = 5.38; found C = 58.21, H = 3.65, N = 4.58, S = 5.40.

X–ray crystallography

X–ray crystal data for complex **1** was collected on a RIGAKU SATURN-724+ CCD single crystal X–ray diffractometer using Mo-Kα radiation. The unit cell determination and data integration were carried out using the CrysAlis package of Oxford Diffraction.[2] The structure was solved by direct methods using OLEX-2 software[3] with the SHELXS structure solution program and refined by full-matrix least-squares on F^2 with SHELXL-97.[4] All H atoms attached to carbon atoms were introduced in idealized positions (d_{CH} = 0.96 Å) using the riding model with their isotropic displacement parameters fixed at 120% of their riding atom. Positional parameters of the H attached to O atom was obtained from difference Fourier syntheses and verified by the geometric parameters of the corresponding hydrogen bonds. Crystal was containing solvent disorder per unit cell; therefore, solvent mask was calculated available in the OLEX-2 program which removed the disorder of guest solvents (CH_2Cl_2) and reduced the value of R_{1obs} from 0.0776 to 0.0598. Using solvent mask, 85.3 electrons were found in the volume of $447a^3$ in one void per unit cell which was consistent to the presence of one CH_2Cl_2 per formula unit which accounts for 84.93 electrons per unit cell.

CCDC entry 2189879 (**1**) contains the crystallographic data for this paper. These data can be obtained free of charge from the Cambridge Crystallographic Data Centre (www.ccdc.cam.ac.uk/data_request/cif).

Electrochemistry

Cyclic voltammetric measurements were conducted in CH_3CN with 0.1M $[N(n\text{-}Bu_4)][PF_6]$ (tetrabutylammoniumhexafluorophosphate) as the supporting electrolyte that was dried in vacuum at 383 K. An Autolab potentiostat with a GPES electrochemical interface was used to carry out the cyclic voltammetry measurements. Glassy carbon disc (diameter 3 mm, freshly polished) was chosen as a working electrode for cyclic voltammetry. Platinum wire was used as the counter electrode. The reference electrode was a non-aqueous Ag/Ag^+ electrode (CH Instruments, 0.01 M $AgNO_3$ in CH_3CN). All the potentials are quoted against the ferrocene-ferrocenium couple (Fc/Fc^+); ferrocene was added as an internal standard at the end of the experiments. For electrochemical measurements, all solutions were prepared from dry CH_3CN (Sigma-Aldrich, spectroscopic grade, dried with molecular sieves (MS, 3 Å). For bulk electrolysis experiments, a carbon rod was used as the working electrode with platinum as the counter electrode and non-aqueous Ag/Ag^+ as the reference electrode (CH Instruments, 0.01 M $AgNO_3$ in CH_3CN).

Computational Details

All the DFT calculations are carried out in Turbomole 7.2[5] program suite with Grimme's D3 dispersion[6] correction. Geometry optimization as well as frequency calculations are performed with B3LYP and BP86 exchange-correlation functionals[7-10] along with def2-TZVP basis set[11,12]. Single point energy is calculated in acetonitrile solvent using the gas phase optimized structures. Gibbs Free energy of all these complexes is calculated in acetonitrile solvent (dielectric constant (ϵ) = 37.5) using the COSMO solvation model[13,14] as implemented in the Turbomole software. The redox potential of all the complexes has been calculated relative to Fc/Fc^+ reference electrode in the acetonitrile solvent following the method reported in the previous literatures. [15,16]

References:

[1] (a) M. D. Sampson and C. P. Kubiak, Inorg. Chem., 2015, 54, 6674–6676. (b) G. K. Rao, M. P. Jamshidi, J. I. G. Dawkins, W. Pell, I. Korobkov and D. Richeson, Dalton Trans., 2017, 46, 6518–6522.

[2] CrysAlis RED, Version 1.171.34.76, Oxford Diffraction Ltd, 2003.

[3] O.V. Dolomanov, L. J. Bourhis, R. J. Gildea, J. A. K. Howard and H. J. Puschmann, *Appl. Crystallogr.*, 2009, **42**, 339-341.

[4] G. Sheldrick, *Acta Crystallogr., Sect. A: Fundam. Crystallogr.*, 2008, **64**, 112–122.

[5] Turbomole V7.2 2017, a development of University of Karlsruhe and Forschungszentrum Karlsruhe GmbH, 1989-2007, TURBOMOLE GmbH, since **2007**; available from http://www.turbomole.com.

[6] S. Grimme, J. Antony, S. Ehrlich, H. A. Krieg, *J. Chem. Phys.* **2010**, *132*, 154104.

[7] A. D. Becke, *J. Chem. Phys.* **1993**, *98*, 1372–1377.

[8] C. Lee, W. Yang, R. G. Parr, *Phys. Rev. B* **1988**, *37*, 785–789.

[9] Becke, A. D. *Phys. Rev. A* **1988**, *38*, 3098–3100.

[10] J. P. Perdew, W. Yue, *Phys. Rev. B* **1986**, *33*, 8800–8802.

[11] F. Weigend, M. Häser, H. Patzelt, R. Ahlrichs, *Chem. Phys. Lett.* **1998**, *294*, 143.

[12] F. Weigend, R. Ahlrichs, *Phys. Chem. Chem. Phys.* **2005**, *7*, 3297–3305.

[13] A. Klamt and G. Schüürmann, *J. Chem. Soc. Perkin Trans*. **1993**, *2*, 799–805.

[14] A. Schäfer, A. Klamt, D. Sattel, J. C. W. Lohrenz, F. Eckert, *Phys. Chem. Chem. Phys.* **2000**, *2*, 2187–2193.

[15] M. Natarajan, H. Faujdar, S. M. Mobin, M. Stein, S. Kaur-Ghumaan, *Dalton Trans.* **2017**, *46*, 10050–10056.
[16] A. V. Marenich, J. M. Ho, M. L. Coote, C. J. Cramer, D. G. Truhlar, *Phys. Chem. Chem. Phys.* **2014**, *16*, 15068–15106.

Scheme S1. ECEC reaction mechanism of proton reduction of complexes **1** and **3** (on the left) and complexes **2** and **4** (on the right).

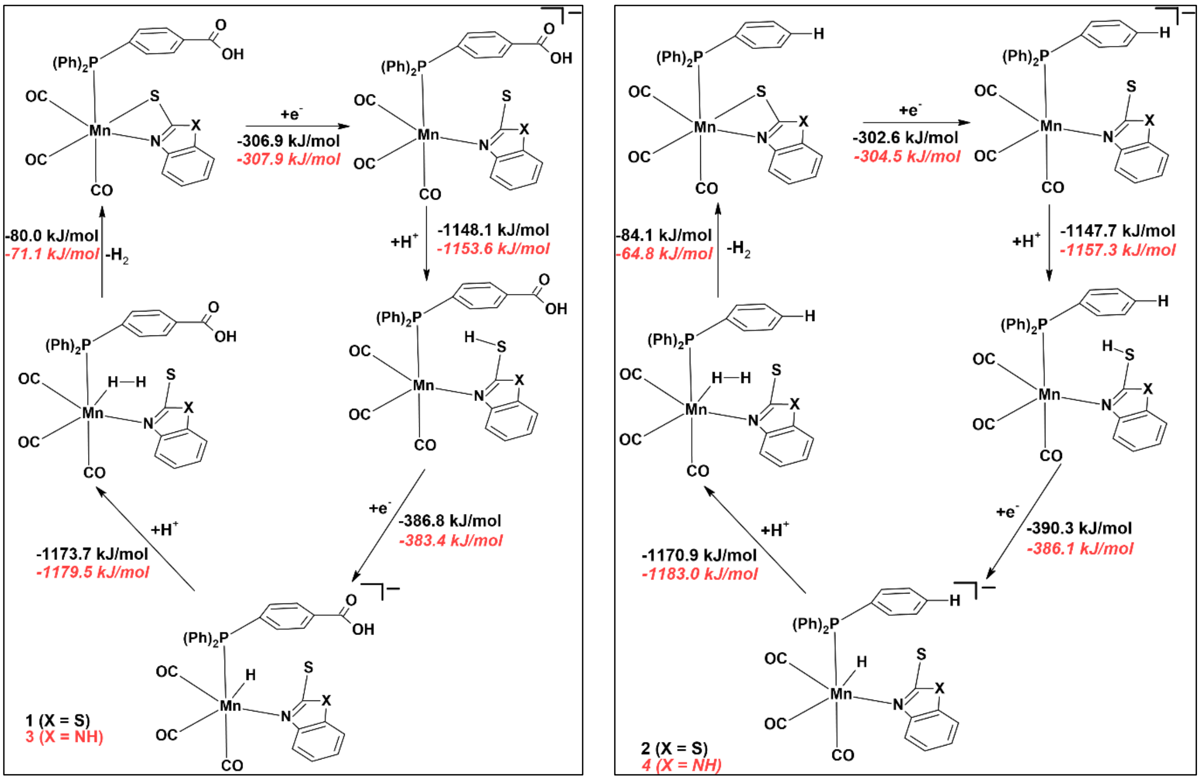

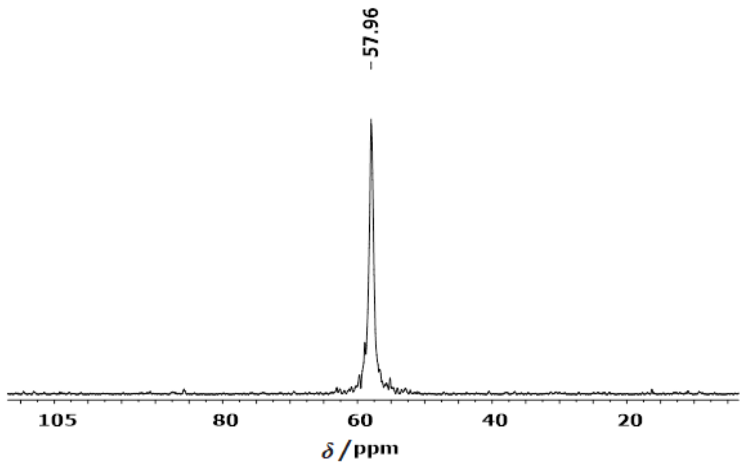

Figure S1. ^{31}P {^{1}H} NMR spectrum for complex **1** (161.8 MHz, $CDCl_3$, 298 K).

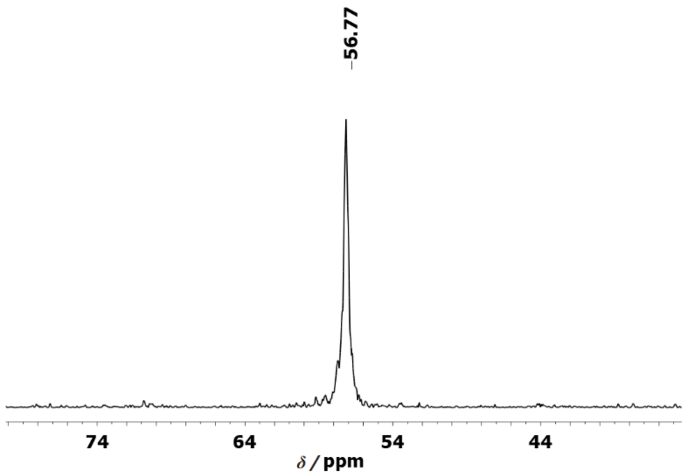

Figure S2. ^{31}P {^{1}H} NMR spectrum for complex **2** (161.8 MHz, $CDCl_3$, 298 K).

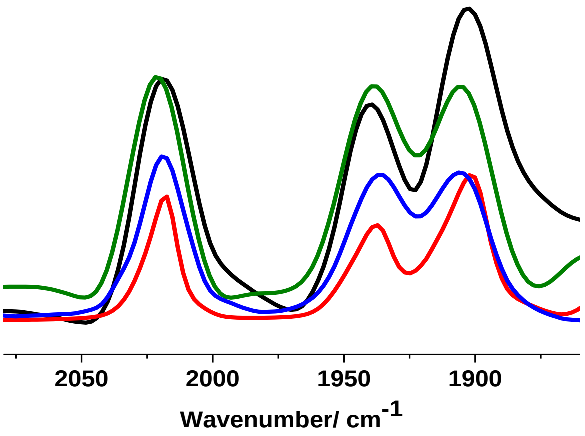

Figure S3. FTIR spectra for complexes **1** (—), **2** (—), **3** (—) and **4** (—) in dichloromethane.

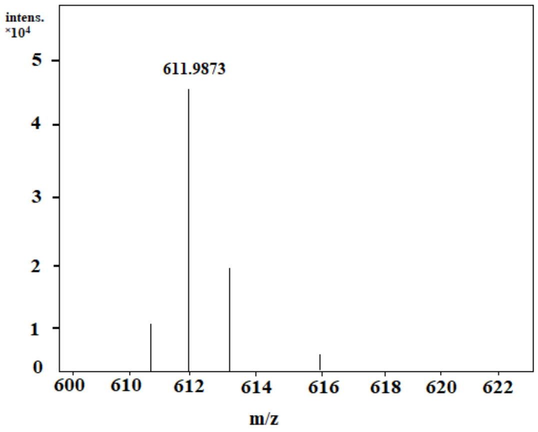

Figure S4. Mass spectrum in CH_3CN for complex **1**. *m/z*: [**M**+H]$^+$ peak at 611.98.

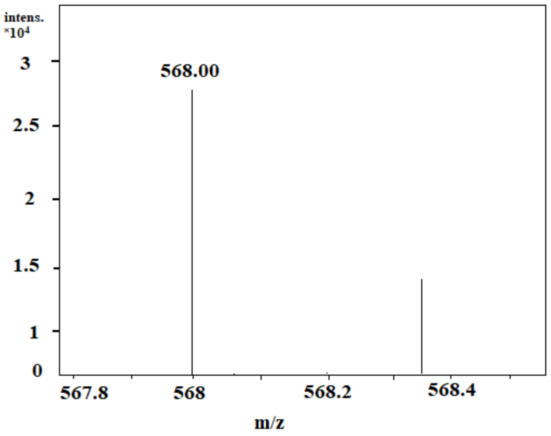

Figure S5. Mass spectrum in CH_3CN for complex **2**. *m/z*: [**M**]$^+$ peak at 568.

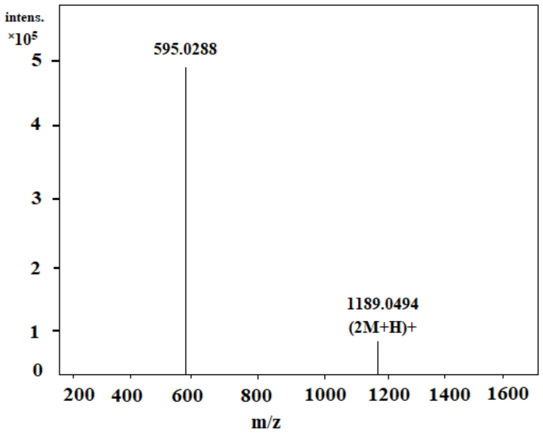

Figure S6. Mass spectrum in CH_3CN for complex **3**. *m/z*: [**M**+H]$^+$ peak at 595.02.

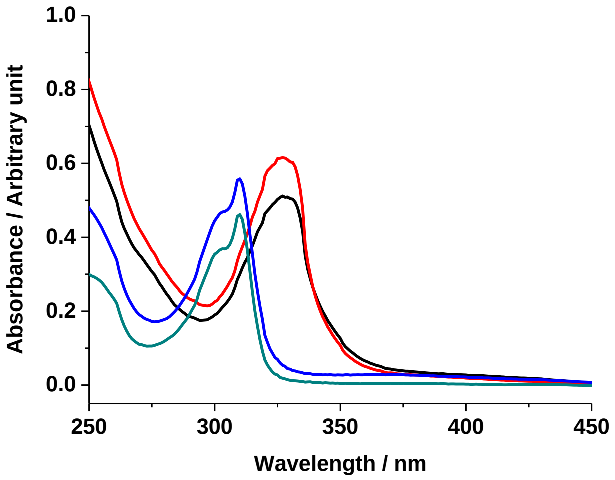

Figure S7. UV-Vis absorption spectra for complexes **1** (—), **2** (—), **3** (—) and **4** (—) in CH_3CN.

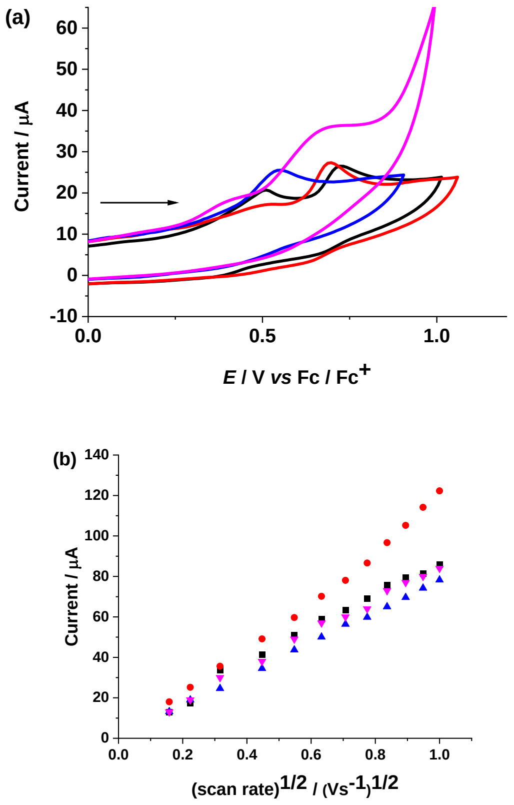

Figure S8. (a) Cyclic voltammograms for oxidation of complexes **1** (—), **2** (—), **3** (—) and **4** (—) (1 mM) in CH_3CN at a scan rate of 0.1 Vs^{-1}. (b) Plots of current *vs.* (scan rate)$^{1/2}$ (0.1–1 Vs^{-1}) for the reduction peaks of complexes **1** (■), **2** (●), **3** (▼) and **4** (▲) (1 mM).

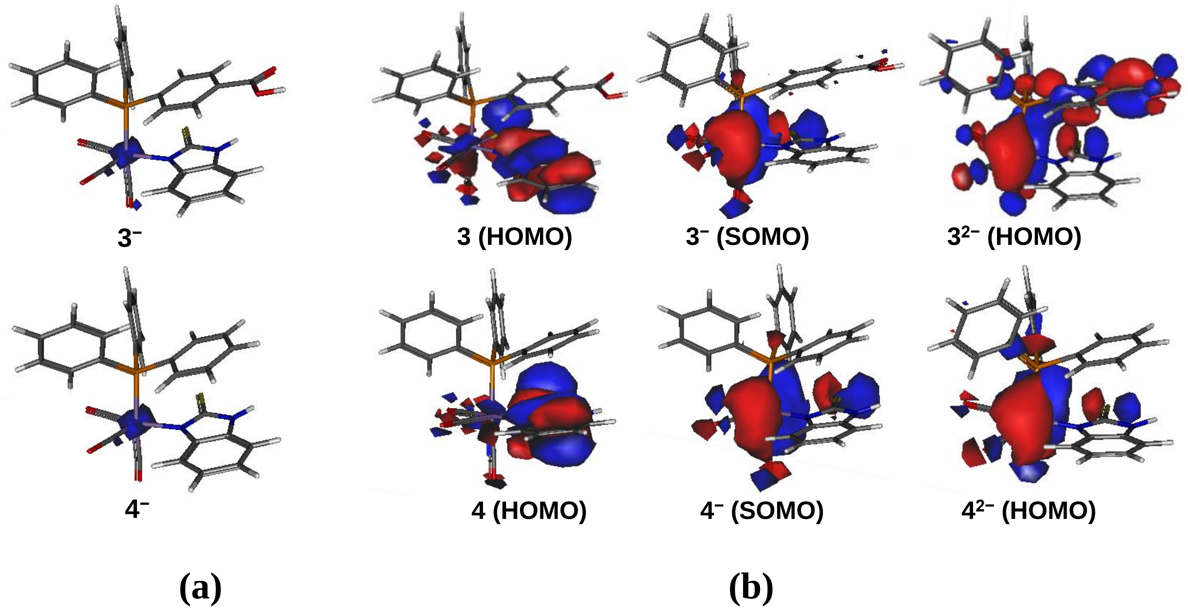

Figure S9. Display of (a) spin density distribution (at an isocontour value = 0.006e) and (b) highest occupied molecular orbitals of complexes **3** and **4** (at an isocontour value = 0.02e). The PhCOOH-*p* group in **1** affords a ligand-based second reduction event $\mathbf{3}^{-}/\mathbf{3}^{2-}$ whereas $\mathbf{4}^{-}/\mathbf{4}^{2-}$ is metal-centered.

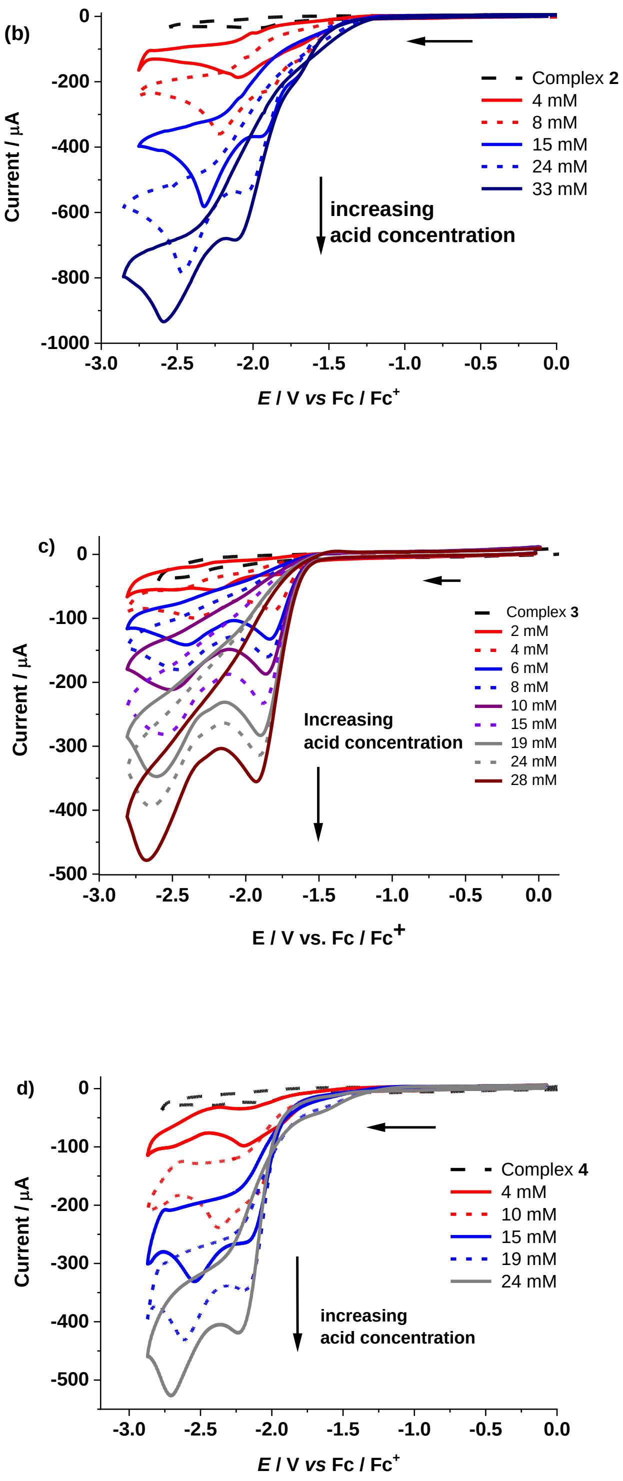

Figure S10. CVs for complexes (a) **2**; (b) **3**; and (c) **4** (1 mM) in the absence (top curve, dashed black line) and presence of TFA (4-33 mM, **2**); (2-28 mM, **3**) and (4-24 mM, **4**) at 0.1 Vs^{-1}.

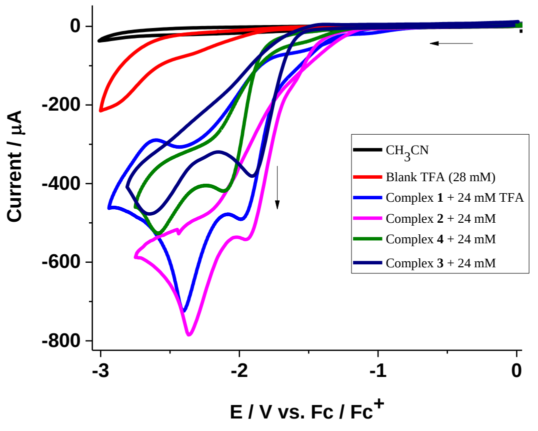

Figure S11. Cyclic voltammograms for blank TFA and complexes (1 mM) **1-4** with TFA in CH_3CN at 0.1 Vs^{-1}.

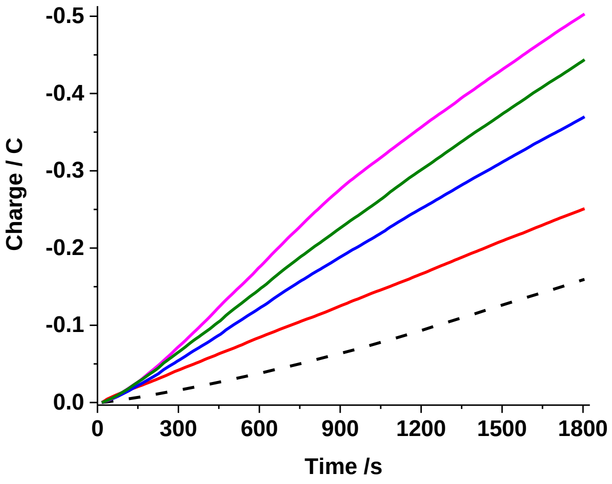

Figure S12. Plots of charge *vs.* time for controlled-potential electrolysis of blank TFA (----, 6 mM) complexes **1** (red), **2** (blue), **3** (green) and **4** (pink) (0.25 mM) between –2 to –2.25 V in CH_3CN / 0.1 M $[N(n\text{-}Bu_4)][PF_6]$ / 6 mM (20 eq) TFA.

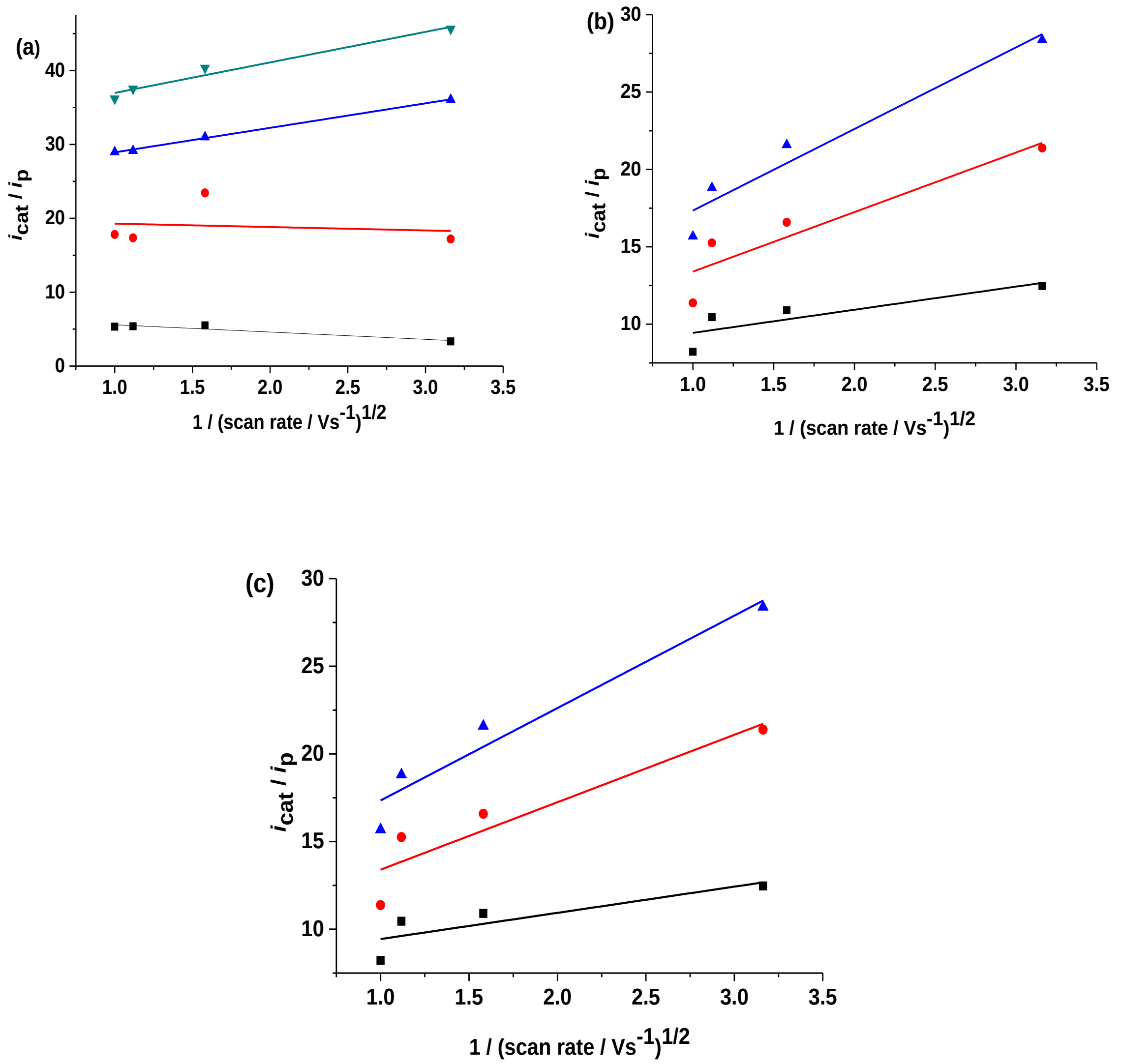

Figure S13. Plots of *of* i_{cat} / i_p *vs.* 1/(scan rate)$^{1/2}$ (1/($Vs^{-1})^{1/2}$) for complexes (1 mM) (a) **1** with 10 mM (■), 37 mM (●), 70 mM (▲) and 96 mM (▼); (b) **2** with 19 mM (■), 37 (●) and 54 mM (▲) and (c) **4** with 19 mM (■), 37 mM (●) and 54 mM (▲) TFA in 0.1 M [N(n-Bu$_4$)][PF$_6$] / CH_3CN. Lines are best fit lines to the data.

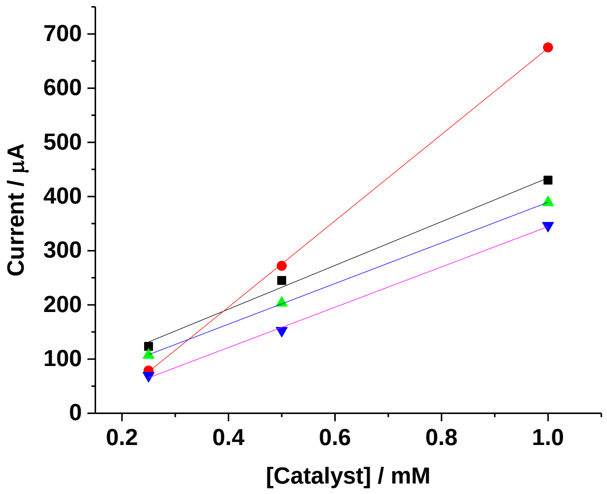

Figure S14. Dependence of i_{cat} on the catalyst concentration for catalyst **1** (■), **2** (●), **3** (▲) and **4** (▼) in the presence of 20 eq (19 mM) TFA. Lines are best fit lines to the data. The negative sign for i_{cat} has been ignored.

Table S1. Crystallographic parameters and refinement details for complex **1**.

**Properties**	**1**
**Empirical formula**	$C_{29}H_{19}MnNO_5PS_2$
**Formula weight**	611.48
**Crystal system**	Monoclinic
**Space group**	$P2_1/n$
**a [Å]**	12.4465(5)
**b [Å]**	14.8434(5)
**c [Å]**	19.2326(7)
**α [°]**	90
**β [°]**	105.920(4)
**γ [°]**	90
**V [Å3]**	3416.9(2)
**Z**	4
**ρ_{calcd}. [mg m^{-3}]**	1.189
**μ (MoK_{α}) [mm^{-1}]**	0.588
**F (000)**	1248.0
**θ range [°]**	6.838 to 51.36
**hkl indices**	±15 ± 18 ± 23
**(R_{int})**	6479 (0.0520)
**R_1, wR_2 [I>2σ(I)]**	0.0598, 0.1488
**R_1, wR_2 (all data)**	0.0868, 0.1646
**$\Delta\rho_{max., min.}$ [e$Å^{-3}$]**	0.53 and –0.27

[a] $R_1 = \Sigma I\ I F_o I - I F c II / \Sigma I F_o I$

[b] $wR_2 = \{\Sigma[w(F^2_o - F^2_c)^2] / \Sigma w(F^2_o)^2\}^{1/2}$

Table S2. Comparison of experimental and computed bond lengths (Å) for complex **1** at BP86 / def2-TZVP and B3LYP / def2-TZVP level of theory.

**1**	**Exp**	**BPD3**	**B3LYP**
**Mn–S**	2.456(13)	2.489	2.526
**Mn–P**	2.366(10)	2.372	2.431
**Mn–N**	2.072(3)	2.058	2.086
**Mn–C$_{(CO)}$**	1.818(4)	1.810	1.835
**Mn–C$_{(CO)}$**	1.805(5)	1.781	1.813
**Mn–C$_{(CO)}$**	1.800(5)	1.791	1.803

Table S3. Bond lengths of complex **1** and of its reaction intermediates formed in HER.

Bond Distances (Å)	1	1^{-}	1SH	$1MnH^{-}$	$1MnH_2$	1^{2-}
			BP86			
**Mn–N**	2.058	2.100	2.093	2.139	2.110	2.139
**Mn–S**	2.489	3.237	3.298	3.633	3.510	3.546
**Mn–P**	2.372	2.299	2.327	2.287	2.370	2.262
**Mn–CO**	1.781–1.810	1.777–1.805	1.780–1.810	1.760–1.798	1.780–1.811	1.757–1.807
**C–O**	1.157–1.164	1.168–1.179	1.161–1.170	1.164–1.177	1.157–1.164	1.180–1.190
**Mn–H1/H2**			2.094	1.583	1.664/2.051	
**S–H1/H2**			1.447	2.476	2.684/1.762	
**H1–H2**					0.932	
			B3LYP			
**Mn–N**	2.086	2.145	2.145	2.197	2.140	2.245
**Mn–S**	2.526	3.369	3.394	3.699	3.551	3.682
**Mn–P**	2.431	2.349	2.373	2.337	2.443	2.238
**Mn–CO**	1.803–1.835	1.806–1.820	1.796–1.825	1.771–1.813	1.812–1.840	1.761–1.815
**C–O**	1.141–1.148	1.153–1.164	1.148–1.157	1.149–1.161	1.139–1.146	1.168–1.179
**Mn–H1/H2**			2.286	1.574	1.714/1.890	
**S–H1/H2**			1.390	2.649	2.852/2.052	
**H1–H2**					0.826	

Table S4. Bond lengths of complex **2** and of its reaction intermediates formed in HER.

Bond Distances (Å)	2	2^{-}	2SH	$2MnH^{-}$	$2MnH_2$	2^{2-}
			BP86			
**Mn–N**	2.059	2.095	2.083	2.130	2.116	2.173
**Mn–S**	2.487	3.299	3.277	3.664	3.524	3.658
**Mn–P**	2.364	2.310	2.344	2.304	2.369	2.224
**Mn–CO**	1.787–1.811	1.785–1.804	1.780–1.809	1.761–1.796	1.778–1.810	1.753–1.800
**C–O**	1.164–1.157	1.169–1.178	1.162–1.169	1.166–1.174	1.158–1.164	1.183–1.194
**Mn–H1/H2**			2.057	1.580	1.654/2.134	
**S–H1/H2**			1.462	2.620	2.640/1.675	
**H1–H2**					0.970	
			B3LYP			
**Mn–N**	2.087	2.139	2.136	2.184	2.138	2.270
**Mn–S**	2.523	3.390	3.367	3.708	3.554	3.730
**Mn–P**	2.419	2.361	2.392	2.354	2.448	2.234
**Mn–CO**	1.804–1.835	1.788–1.820	1.796–1.824	1.772–1.810	1.811–1.838	1.758–1.807
**C–O**	1.141–1.148	1.154–1.164	1.149–1.153	1.150–1.159	1.140–1.147	1.170–1.182
**Mn–H1/H2**			2.251	1.575	1.712/1.895	
**S–H1/H2**			1.398	2.754	2.842/2.042	
**H1–H2**					0.827	

Table S5. Bond lengths of complex **3** and of its reaction intermediates formed in HER.

Bond Distances (Å)	3	3^-	3SH	$3MnH^-$	$3MnH_2$	3^{2-}
			**BP86**			
**Mn–N**	2.059	2.069	2.073	2.103	2.076	2.109
**Mn–S**	2.520	3.438	3.383	3.734	3.561	3.670
**Mn–P**	2.361	2.309	2.331	2.292	2.373	2.261
**Mn–$C_{(CO)}$**	1.786–1.812	1.775–1.807	1.780–1.809	1.764–1.796	1.787–1.813	1.760–1.808
**C–O**	1.157–1.165	1.168–1.178	1.162–1.170	1.164–1.174	1.156–1.163	1.180–1.190
**Mn–H1/H2**			2.179	1.578	1.682/1.989	
**S–H1/H2**			1.441	2.599	2.764/1.889	
**H1–H2**					0.894	
			**B3LYP**			
**Mn–N**	2.084	2.110	2.120	2.263	2.099	2.176
**Mn–S**	2.559	3.554	3.512	3.877	3.622	3.765
**Mn–P**	2.415	2.356	2.378	2.344	2.443	2.251
**Mn–$C_{(CO)}$**	1.810–1.837	1.789–1.822	1.795–1.824	1.780–1.815	1.815–1.839	1.770–1.816
**C–O**	1.141–1.148	1.153–1.164	1.149–1.157	1.150–1.158	1.139–1.146	1.167–1.179
**Mn–H1/H2**			2.440	1.610	1.723/1.888	
**S–H1/H2**			1.379	3.044	2.908/2.118	
**H1–H2**					0.817	

Table S6. Bond lengths of complex **4** and of its reaction intermediates formed in HER.

Bond Distances (Å)	4	4^{-}	4SH	$4MnH^{-}$	$4MnH_2$	4^{2-}
			BP86			
Mn–N	2.057	2.074	2.068	2.095	2.083	2.139
Mn–S	2.522	3.511	3.361	3.744	3.572	3.786
Mn–P	2.363	2.311	2.336	2.302	2.370	2.215
$Mn–C_{(CO)}$	1.786–1.811	1.774–1.804	1.793–1.808	1.764–1.796	1.781–1.810	1.755–1.801
C–O	1.157–1.165	1.169–1.179	1.163–1.170	1.166–1.175	1.157–1.165	1.184–1.194
Mn–H1/H2			2.145	1.579	1.663/2.137	
S–H1/H2			1.452	2.693	2.661/1.717	
H1–H2					0.950	
			B3LYP			
Mn–N	2.079	2.109	2.116	2.141	2.099	2.211
Mn–S	2.561	3.582	3.491	3.787	3.617	3.831
Mn–P	2.415	2.357	2.382	2.346	2.449	2.224
$Mn–C_{(CO)}$	1.804–1.836	1.788–1.820	1.794–1.823	1.775–1.810	1.813–1.838	1.760–1.805
C–O	1.141–1.149	1.154–1.164	1.150–1.156	1.150–1.159	1.140–1.147	1.171–1.181
Mn–H1/H2			2.421	1.573	1.720/1.898	
S–H1/H2			1.383	2.777	2.886/2.094	
H1–H2					0.820	

Table S7. Reduction Potential (RP, in V) calculated at BP86 / def2-TZVP (B3LYP / def2-TZVP) level of theory.

Complex	1st one-electron RP	2nd one-electron RP	Two-electron RP
	$1 + e^{-} \rightarrow 1^{-}$	$1^{-} + e^{-} \rightarrow 1^{2-}$	$1 + 2e^{-} \rightarrow 1^{2-}$
**1**	–1.63 (–1.33)	–2.16 (–2.12)	–1.89 (–1.72)
**2**	–1.67 (–1.36)	–2.18 (–2.19)	–1.92 (–1.77)
**3**	–1.62 (–1.30)	–2.20 (–2.17)	–1.91 (–1.74)
**4**	–1.65 (–1.31)	–2.19 (–2.18)	–1.92 (–1.74)

Table S8. Energy and free energy change (ΔE and ΔG, in kJ/mol) in HER of complex **1**.

Reaction	BP (B3LYP)			
	**ΔE _in gas**	**ΔG _in gas**	**ΔE_in solvent**	**ΔG_in solvent**
**$1 \rightarrow 1^{-}$**	–154.5 (–126.2)	–166.1 (–138.9)	–295.4 (–269.7)	–306.9 (–282.4)
**$1^{-} \rightarrow$ 1SH**	–1310.5 (–1316.6)	–1285.7 (–1290.1)	–1172.9 (–1178.6)	–1148.1 (–1152.1)
**1SH $\rightarrow$ $1MnH^{-}$**	–245.4 (–219.2)	–249.0 (–223.1)	–383.2 (–357.1)	–386.8 (–361.0)
**$1MnH^{-} \rightarrow 1MnH_2$**	–1336.1 (–1355.0)	–1314.5 (–1325.0)	–1195.3 (–1217.5)	–1173.7 (–1187.5)
**$1MnH_2 \rightarrow 1 + H_2$**	–41.8 (–59.4)	–79.4 (–105.1)	–42.4 (–54.4)	–80.0 (–100.2)
**$1^{-} \rightarrow 1^{2-}$**	129.5 (192.3)	114.2 (179.1)	–240.6 (–193.1)	–255.9 (–206.3)
**$1^{2-} \rightarrow 1MnH^{-}$**	–1685.4 (–1728.1)	–1648.9 (–1692.3)	–1315.5 (–1342.6)	–1279.0 (–1306.8)

Table S9. Energy and free energy change (ΔE and ΔG, in kJ/mol) in HER of complex **2**.

Reaction	BP (B3LYP)			
	**ΔE _in gas**	**ΔG _in gas**	**ΔE_in solvent**	**ΔG_in solvent**
**$2 \rightarrow 2^{-}$**	–140.1 (–115.3)	–155.5 (–130.6)	–287.2 (–264.3)	–302.6 (–279.6)
**$2^{-} \rightarrow$ 2SH**	–1315.8 (–1320.1)	–1290.3 (–1298.5)	–1173.2 (–1177.9)	–1147.7 (–1156.3)
**2SH $\rightarrow$ $2MnH^{-}$**	–245.6 (–221.4)	–248.7 (–219.0)	–387.2 (–362.9)	–390.3 (–360.5)
**$2MnH^{-} \rightarrow 2MnH_2$**	–1343.6 (–1359.9)	–1317.2 (–1329.3)	–1197.2 (–1216.8)	–1170.9 (–1186.2)
**$2MnH_2 \rightarrow 2 + H_2$**	–43.2 (–59.8)	–83.0 (–104.8)	–44.4 (–55.5)	–84.1 (–100.4)
**$2^{-} \rightarrow 2^{2-}$**	158.9 (213.1)	144.1 (203.1)	–239.5 (–189.7)	–254.3 (–199.7)
**$2^{2-} \rightarrow 2MnH^{-}$**	–1720.3 (–1754.6)	–1683.1 (–1720.6)	–1320.9 (–1351.1)	–1283.7 (–1317.1)

Table S10. Energy and free energy change (ΔE and ΔG, in kJ/mol) in HER of complex **3**.

**Reaction**	**BP (B3LYP)**			
	**ΔE _in gas**	**ΔG _in gas**	**ΔE_in solvent**	**ΔG_in solvent**
**3 → 3^{-}**	–155.0 (–131.2)	–168.9 (–143.0)	–294.1 (–273.2)	–307.9 (–285.0)
**3^{-} → 3SH**	–1316.6 (–1320.1)	–1286.4 (–1291.9)	–1183.8 (–1188.3)	–1153.6 (–1160.0)
**3SH → $3MnH^{-}$**	–251.3 (–231.5)	–251.6 (–230.2)	–383.1 (–362.1)	–383.4 (–360.8)
**$3MnH^{-}$ → $3MnH_2$**	–1340.2 (–1354.6)	–1319.1 (–1331.6)	–1200.6 (–1216.3)	–1179.5 (–1193.3)
**$3MnH_2$ → 3 + H_2**	–25.2 (–39.0)	–68.8 (–85.5)	–27.5 (–37.5)	–71.1 (–84.0)
**3^{-} → 3^{2-}**	128.7 (194.6)	119.4 (184.4)	–242.5 (–191.4)	–251.8 (–201.6)
**3^{2-} → $3MnH^{-}$**	–1696.5 (–1746.2)	–1657.3 (–1706.5)	–1324.5 (–1358.9)	–1285.3 (–1319.2)

Table S11. Energy and free energy change (ΔE and ΔG, in kJ/mol) in HER of complex **4**.

**Reaction**	**BP (B3LYP)**			
	**ΔE _in gas**	**ΔG _in gas**	**ΔE_in solvent**	**ΔG_in solvent**
**4 → 4^{-}**	–142.9 (–119.6)	–157.5 (–134.7)	–289.9 (–269.2)	–304.5 (–284.2)
**4^{-} → 4SH**	–1322.5 (–1326.0)	–1296.5 (–1300.2)	–1183.2 (–1188.9)	–1157.3 (–1163.0)
**4SH → $4MnH^{-}$**	–247.2 (–222.2)	–249.1 (–223.1)	–384.2 (–357.4)	–386.1 (–358.3)
**$4MnH^{-}$ → $4MnH_2$**	–1351.4 (–1369.7)	–1329.5 (–1340.7)	–1204.8 (–1224.4)	–1183.0 (–1195.4)
**$4MnH_2$ → 4 + H_2**	–24.3 (–38.9)	–62.0 (–83.5)	–27.1 (–37.5)	–64.8 (–82.1)
**4^{-} → 4^{2-}**	160.3 (216.5)	149.4 (206.1)	–241.7 (–190.5)	–252.5 (–200.9)
**4^{2-} → $4MnH^{-}$**	–1730.0 (–1764.7)	–1695.1 (–1729.4)	–1325.7 (–1355.8)	–1290.8 (–1320.4)
