# Supplementary material for: Mononuclear manganese complexes as hydrogen evolving catalysts
Source: Front Chem. 2022 Oct 7;10:993085. doi: 10.3389/fchem.2022.993085 (PMC9585328; doi:10.3389/fchem.2022.993085)

checkCIF/PLATON report

Structure factors have been supplied for datablock(s) vk-41a

THIS REPORT IS FOR GUIDANCE ONLY. IF USED AS PART OF A REVIEW PROCEDURE FOR PUBLICATION, IT SHOULD NOT REPLACE THE EXPERTISE OF AN EXPERIENCED CRYSTALLOGRAPHIC REFEREE.

No syntax errors found. CIF dictionary Interpreting this report

Datablock: vk-41a

Bond precision: C-C = 0.0068 A Wavelength=0.71073

Cell:	a=12.4465(5)	b=14.8434(5)	c=19.2326(7)
	alpha=90	beta=105.920(4)	gamma=90
Temperature:	298 K		

	Calculated	Reported
Volume	3416.9(2)	3416.9(2)
Space group	P 21/n	P 1 21/n 1
Hall group	-P 2yn	-P 2yn
Moiety formula	C29 H19 Mn N O5 P S2 [+ solvent]	C29 H19 Mn N O5 P S2
Sum formula	C29 H19 Mn N O5 P S2 [+ solvent]	C29 H19 Mn N O5 P S2
Mr	611.48	611.48
Dx,g cm-3	1.189	1.189
Z	4	4
Mu (mm-1)	0.588	0.588
F000	1248.0	1248.0
F000'	1251.22	
h,k,lmax	15,18,24	15,18,24
Nref	6982	6965
Tmin,Tmax	0.946,0.951	0.704,1.000
Tmin'	0.946	

Correction method= # Reported T Limits: Tmin=0.704 Tmax=1.000
AbsCorr = MULTI-SCAN

Data completeness= 0.998 Theta(max)= 26.371

R(reflections)= 0.0638(4578)

wR2(reflections)= 0.1815(6965)

S = 1.033 Npar= 352

The following ALERTS were generated. Each ALERT has the format
test-name_ALERT_alert-type_alert-level.
Click on the hyperlinks for more details of the test.

Alert level B

```
PLAT230_ALERT_2_B Hirshfeld Test Diff for    S2       --C3       .       10.0 s.u.
PLAT232_ALERT_2_B Hirshfeld Test Diff (M-X)  Mn1      --S1       .       12.2 s.u.
PLAT910_ALERT_3_B Missing # of FCF Reflection(s) Below Theta(Min).         15 Note
```

Alert level C

```
CRYSC01_ALERT_1_C The word below has not been recognised as a standard
            identifier.
            yellowish
PLAT094_ALERT_2_C Ratio of Maximum / Minimum Residual Density ....       2.44 Report
PLAT230_ALERT_2_C Hirshfeld Test Diff for    C5       --C6       .        6.0 s.u.
PLAT241_ALERT_2_C High   'MainMol' Ueq as Compared to Neighbors of         S1 Check
PLAT241_ALERT_2_C High   'MainMol' Ueq as Compared to Neighbors of         S2 Check
PLAT242_ALERT_2_C Low    'MainMol' Ueq as Compared to Neighbors of        C29 Check
PLAT331_ALERT_2_C Small Aver Phenyl C-C Dist C17      --C22      .       1.37 Ang.
PLAT341_ALERT_3_C Low Bond Precision on  C-C Bonds ...............    0.00676 Ang.
PLAT911_ALERT_3_C Missing FCF Refl Between Thmin & STh/L=    0.600          2 Report
```

Alert level G

```
PLAT007_ALERT_5_G Number of Unrefined Donor-H Atoms ..............          1 Report
PLAT230_ALERT_2_G Hirshfeld Test Diff for    O2       --C9       .        5.5 s.u.
PLAT232_ALERT_2_G Hirshfeld Test Diff (M-X)  Mn1      --C9       .        7.0 s.u.
PLAT232_ALERT_2_G Hirshfeld Test Diff (M-X)  Mn1      --C10      .        7.3 s.u.
PLAT606_ALERT_4_G Solvent Accessible VOID(S) in Structure ........          ! Info
PLAT883_ALERT_1_G No Info/Value for _atom_sites_solution_primary .     Please Do !
PLAT933_ALERT_2_G Number of HKL-OMIT Records in Embedded .res File          2 Note
PLAT978_ALERT_2_G Number C-C Bonds with Positive Residual Density.          2 Info
```

---

0 **ALERT level A** = Most likely a serious problem - resolve or explain
3 **ALERT level B** = A potentially serious problem, consider carefully
9 **ALERT level C** = Check. Ensure it is not caused by an omission or oversight
8 **ALERT level G** = General information/check it is not something unexpected

2 ALERT type 1 CIF construction/syntax error, inconsistent or missing data
13 ALERT type 2 Indicator that the structure model may be wrong or deficient
3 ALERT type 3 Indicator that the structure quality may be low
1 ALERT type 4 Improvement, methodology, query or suggestion
1 ALERT type 5 Informative message, check

It is advisable to attempt to resolve as many as possible of the alerts in all categories. Often the minor alerts point to easily fixed oversights, errors and omissions in your CIF or refinement strategy, so attention to these fine details can be worthwhile. In order to resolve some of the more serious problems it may be necessary to carry out additional measurements or structure refinements. However, the purpose of your study may justify the reported deviations and the more serious of these should normally be commented upon in the discussion or experimental section of a paper or in the "special_details" fields of the CIF. checkCIF was carefully designed to identify outliers and unusual parameters, but every test has its limitations and alerts that are not important in a particular case may appear. Conversely, the absence of alerts does not guarantee there are no aspects of the results needing attention. It is up to the individual to critically assess their own results and, if necessary, seek expert advice.

Publication of your CIF in IUCr journals

A basic structural check has been run on your CIF. These basic checks will be run on all CIFs submitted for publication in IUCr journals (*Acta Crystallographica*, *Journal of Applied Crystallography*, *Journal of Synchrotron Radiation*); however, if you intend to submit to *Acta Crystallographica Section C* or *E* or *IUCrData*, you should make sure that full publication checks are run on the final version of your CIF prior to submission.

Publication of your CIF in other journals

Please refer to the *Notes for Authors* of the relevant journal for any special instructions relating to CIF submission.

PLATON version of 18/05/2022; check.def file version of 17/05/2022

Datablock vk-41a - ellipsoid plot

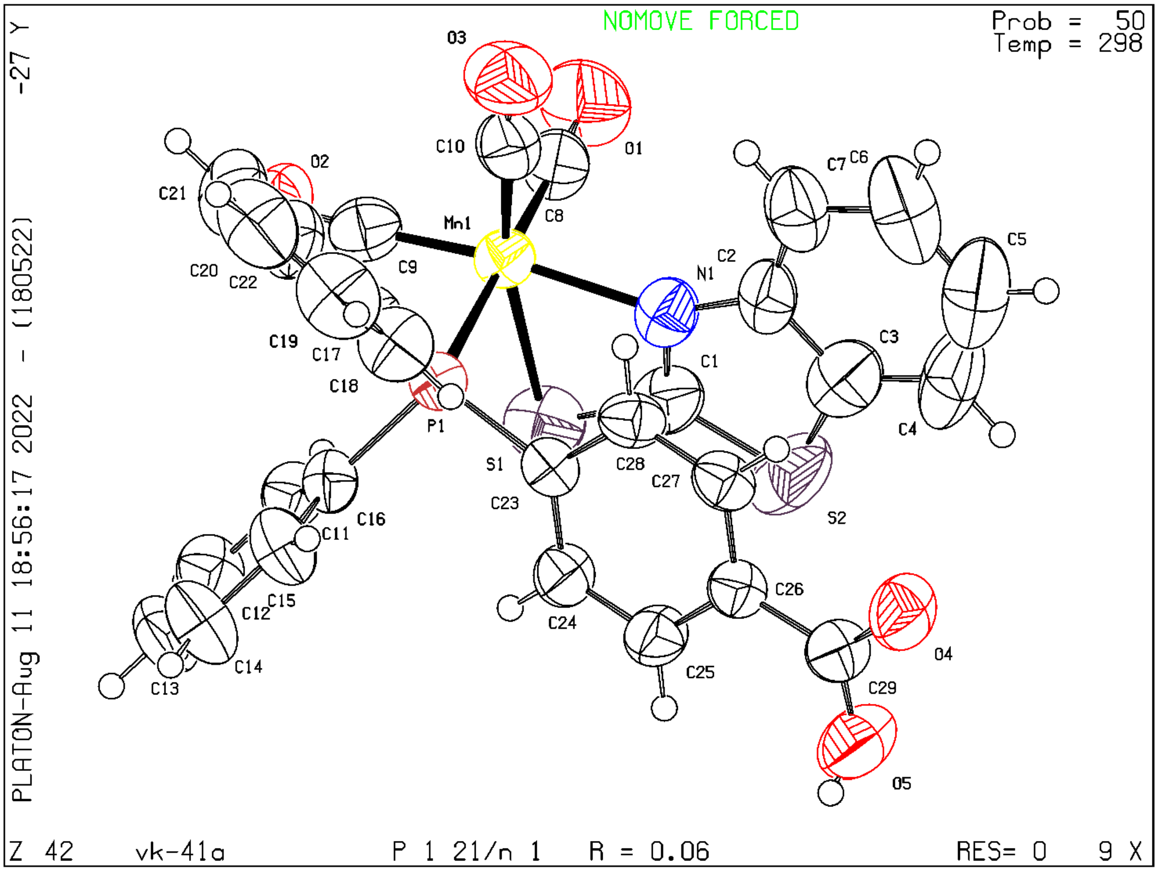

Supplement: Supplementary file 2 [file DataSheet1.pdf]
